# Radiative loss of coherence in free electrons: a long-range quantum phenomenon

**DOI:** 10.1038/s41377-023-01361-6

**Published:** 2024-01-26

**Authors:** Cruz I. Velasco, Valerio Di Giulio, F. Javier García de Abajo

**Affiliations:** 1 ICFO-Institut de Ciencies Fotoniques, The Barcelona Institute of Science and Technology, 08860 Castelldefels (Barcelona), Spain; 2 ICREA-Institució Catalana de Recerca i Estudis Avançats, Passeig Lluís Companys 23, 08010 Barcelona, Spain

**Keywords:** Quantum optics, Quantum optics

## Abstract

Quantum physics rules the dynamics of small objects as they interact over microscopic length scales. Nevertheless, quantum correlations involving macroscopic distances can be observed between entangled photons as well as in atomic gases and matter waves at low temperatures. The long-range nature of the electromagnetic coupling between charged particles and extended objects could also trigger quantum phenomena over large distances. Here, we reveal a manifestation of quantum mechanics that involves macroscopic distances and results in a nearly complete depletion of coherence associated with which-way free-electron interference produced by electron–radiation coupling in the presence of distant extended objects. This is a ubiquitous effect that we illustrate through a rigorous theoretical analysis of a two-path electron beam interacting with a semi-infinite metallic plate and find the inter-path coherence to vanish proportionally to the path separation at zero temperature and exponentially at finite temperature. The investigated regime of large distances originates in the coupling of the electron to radiative modes assisted by diffraction at material structures but without any involvement of material excitations. Besides the fundamental interest of this macroscopic quantum phenomenon, our results suggest an approach to measuring the vacuum temperature and nondestructively sensing the presence of distant objects.

## Abstract (web summary)

Free-electron decoherence produced by electron coupling to radiation constitutes a quantum-physics macroscopic phenomenon that enables nondestructive sensing of distant objects

## Graphical abstract

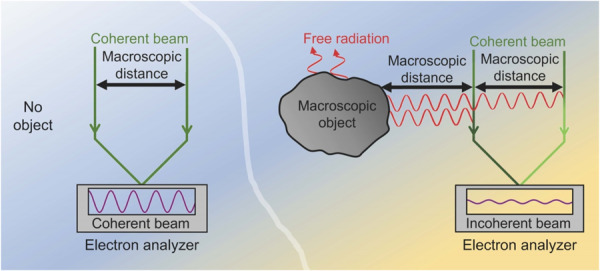

## Introduction

The wave nature of electrons allows us to image materials with atomic resolution in transmission electron microscopy^1,2^ (TEM) and resolve the atomic structure and dynamics of molecules and crystal surfaces through low-energy^3,4^, photoemission^5^, and ultrafast^6,7^ electron diffraction. In these techniques, wave interference takes place between elastically scattered components, while inelastic collisions are typically regarded as a source of decoherence that destroys interference through the addition of a stochastic phaseour results suggest an approach to measuour results suggest an approach to measuour results suggest an approach to measu.

Decoherence can be produced by coupling to material excitations. In particular, an electron split into two paths and moving parallel to a lossy planar surface was proposed^8^, extensively studied from a theoretical viewpoint^8–14^, and experimentally confirmed^15–19^ to be a suitable configuration to observe electron decoherence. In a related scenario, inelastic electron scattering generated by coupling to thermally populated low-energy material excitations was shown to render an observable loss of electron coherence that limits spatial resolution in TEM^20,21^. An extension to decoherence of charged particles trapped near a lossy surface has recently been made^22^.

Electron decoherence is equally produced by inelastic excitations associated with photon emission and electromagnetic vacuum fluctuations, as predicted for an electron prepared in a prescribed two-path configuration^23,24^, including the effect of neighboring perfect-conductor boundaries^9,11,23^. Likewise, radiative electron decoherence is anticipated to take place due to bremsstrahlung emission^25^, interaction with time-varying fields^26^, and the Smith-Purcell effect^27^. Intriguingly, recoherence can occur for electrons moving in a squeezed vacuum^28^.

**Fig. 1:**
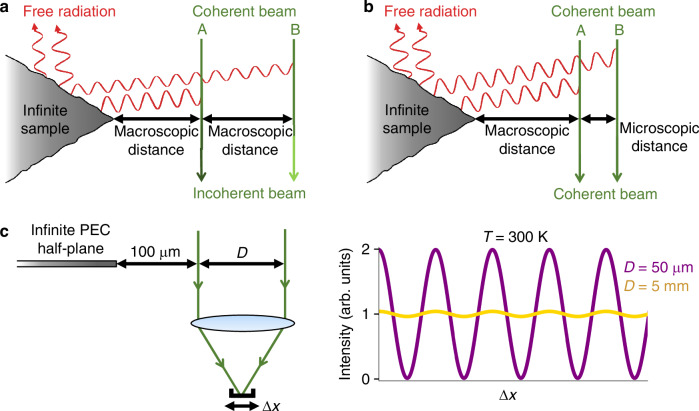
Electron-beam decoherence due to radiative coupling assisted by extended scatterers. **a** An electron split into two paths A and B separated by a large (macroscopic) distance undergoes strong decoherence through coupling to radiation assisted by a distant extended scatterer. **b** For small (microscopic) inter-path separations, coherence is however preserved. **c** We show a specific geometry in which one can substantially vary the degree of decoherence by modifying the inter-path separation for a fixed distance to a perfect-electric-conductor (PEC) half-plane at a temperature of 300 K (see also Fig. S2 in SI), translating into a radical change in the visibility of interference fringes as a function of transverse position Δ*x* at an electron detector (see Fig. S1 in SI)

Decoherence can be intuitively understood through the following analysis for an *incident* electron whose wave function ${\psi }^{{{{\rm{inc}}}}}={\psi }_{{{{\rm{A}}}}}^{{{{\rm{inc}}}}}+{\psi }_{{{{\rm{B}}}}}^{{{{\rm{inc}}}}}$ is split into two non-overlapping paths A and B (Fig. 1a, b). Scattering by a structure produces an overall post-interaction state ${\sum }_{n}(\vert {\psi }_{{{{\rm{A}}}},n}\rangle +\vert {\psi }_{{{{\rm{B}}}},n}\rangle)\otimes \vert n\rangle$, where *n* runs over excitations of the involved materials and the radiation field, while *ψ*_A,*n*_ and *ψ*_B,*n*_ denote the electron wave functions in paths A and B resulting after an excitation *n* is generated. In an interference experiment, electron fringes are formed at a detection plane where the electron paths overlap (Fig. 1c). The amplitude of such fringes $\propto {\sum }_{n}{{{\rm{Re}}}}\{\langle {\psi }_{{{{\rm{A}}}},n}| {\psi }_{{{{\rm{B}}}},n}\rangle \}$ is contributed by wave function components in which the same mode *n* is excited by both paths. A certain degree of coherence is then preserved if paths A and B can both excite a given mode *n* with similar amplitudes, just like in a quantum eraser^29,30^ that produces a loss of which-way information. This is essentially the principle behind inelastic electron holography^31–33^, where interference fringes are observed in energy-filtered inelastically scattered electrons (e.g., after they excite a delocalized plasmon that overlaps both electron paths).

In this analysis, the incident electron is prepared in a pure state characterized by a density matrix $\left\vert {\psi }^{{{{\rm{inc}}}}}\right\rangle \left\langle {\psi }^{{{{\rm{inc}}}}}\right\vert$, while after the interaction, we have a mixed state with a density matrix ${\sum }_{n}(\vert {\psi }_{{{{\rm{A}}}},n}\rangle +\vert {\psi }_{{{{\rm{B}}}},n}\rangle)(\langle {\psi }_{{{{\rm{A}}}},n}\vert +\langle {\psi }_{{{{\rm{B}}}},n}\vert)$ obtained by tracing out material and radiation degrees of freedom, and the resulting loss of visibility in the interference fringes relates to the creation of such inelastic excitations $\left\vert n\right\rangle$. This is conceptually different from diffraction produced after blocking part of the wave function (e.g., in a two-slit experiment), in which the interference fringes are controlled by the shape of the scattering object, but the electron is transmitted in a pure state of wave function ${\psi }^{{{{\rm{tr}}}}}$ and density matrix $\left\vert {\psi }^{{{{\rm{tr}}}}}\right\rangle \left\langle {\psi }^{{{{\rm{tr}}}}}\right\vert$ without the involvement of any excitations. Interestingly, information theory has been invoked to quantitatively separate actual decoherence from elastic diffraction^34^.

In another conceptually different scenario, one can consider electron interactions with classical fields such as those induced by a laser in the context of photon-induced near-field electron microscopy^35^ (PINEM). Here, the electron follows a coherent evolution^36,37^, and therefore, it is characterized by a pure state $\left\vert {\psi }_{{{{\rm{PINEM}}}}}\right\rangle$, even if the electron experiences energy changes (e.g., sidebands in PINEM) and those changes are path-dependent. Consequently, the electron density matrix $\left\vert {\psi }_{{{{\rm{PINEM}}}}}\right\rangle \left\langle {\psi }_{{{{\rm{PINEM}}}}}\right\vert$ remains pure, and different paths can still interfere (e.g., to achieve spatiotemporal electron compression^38^).

In the present work, we are interested in the decoherence produced by the creation of inelastic excitations, and more precisely, radiative modes. Consequently, we consider configurations in which the electron paths do not physically intersect any material (Fig. 1a, b), and the electron–boundary distances are sufficiently large to neglect inelastic excitations created inside the material (see below). This is conceptually different from previous investigations for an aloof electron moving parallel to a planar interface, which leads to decoherence by generating material excitations^8–14^ as well as an elastic phase due to image interactions even for perfect conductors^23,39^.

Because the loss of coherence relates to the different excitation amplitudes associated with each of the electron paths, it is pertinent to recall that the probability that a moving electron undergoes inelastic energy exchanges when passing near an extended material structure presents an infrared divergence due to the contribution of radiative modes^40^, although we anticipate that such a divergence does not lead to any relevant physical pathology as the total energy loss and the degree of coherence remain both finite [see Supplementary Section S5 in the Supplementary Information (SI)]. The results summarized in Table 1 show that divergences are found when free electrons couple to extended material structures, for which the loss probability scales as Γ(*ω*) ∝ 1/*ω* and ∝ 1/*ω*^2^ with the energy loss *ℏω* at zero and finite temperatures, respectively. We show in this work that these divergences produce a radical depletion of coherence for electron paths separated by large distances (i.e., when one of the paths is more exposed to the noted divergences), but a finite degree of coherence is always preserved, and full coherence is recovered as the path separation is reduced. Such preservation of coherence is a key element in off-axis electron holography^41,42^, which relies on interference between electrons passing either through or outside a material to reconstruct its atomic structure.

**Table 1:** Divergence in the spectrally resolved electron energy-loss probability

	

For finite objects (left), the loss probability Γ(*ω*) vanishes at low frequencies as they become increasingly small compared to the light wavelength. We have Γ(*ω*) ∝ *ω*^3^ and ∝ *ω* contributions arising from radiative and nonradiative losses at zero temperature, while an additional factor of 1/*ω* appears at finite temperature *T* because of the scaling of the inelastic-scattering probability as 2*n*_*T*_(*ω*) + 1 ≈ 2*k*_B_*T*/ℏ*ω* + 1, where *n*_*T*_(*ω*) is the Bose-Einstein distribution function. The interaction with a structure that is infinitely extended in a transverse direction with respect to the e-beam (right) produces a divergence as Γ(*ω*) ∝ 1/*ω* at *T* = 0 and ∝ 1/*ω*^2^ at finite *T*

A two-path electron in which one of the paths is close to an extended structure should be a good example to observe a large degree of electron decoherence produced by radiative coupling. We thus consider the configurations depicted in Fig. 1a, b, and indeed, based on the rigorous theory presented below, we obtain a substantial increase in decoherence at room temperature when one of the paths is placed 100 *μ*m away from the edge of a perfect-electric-conductor (PEC) half-plane and the other path is separated by a distance of either 50 *μ*m or 5 mm. This effect can be visualized through the interference fringes formed when the two paths are recombined (see Fig. S1 in SI), as we show in Fig. 1c. A similar effect is observed while maintaining a large inter-path distance (a few mm) by placing a half-plane close (e.g., 1 *μ*m) or far (10 mm) from the nearest electron path (see Fig. S2 in SI). These are situations in which quantum-mechanical effects (decoherence) take place over large distances, a territory that was so far reserved to the lossless propagation of photons in free space^43,44^ or superpositions of matter states at low temperatures^45,46^.

**Fig. 2:**
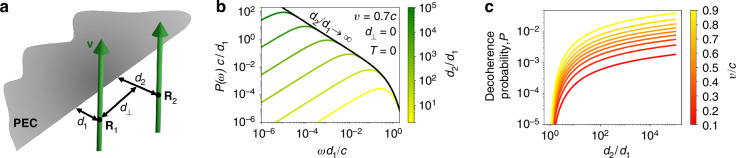
Two-path decoherence by a half-plane at zero temperature. **a** System under consideration, consisting of a single electron split into a two-path spatial superposition and passing outside and perpendicularly to a perfectly conducting half-plane at distances *d*_1_ and *d*_2_ from the edge [beam path positions **R**_1_ = ( *d*_1_,0) and **R**_2_ = (*d*_2_, *d*_⊥_ )]. **b** Universal plot of the spectrally resolved decoherence probability for various *d*_2_/*d*_1_ ratios (colored curves) with *d*_⊥_ = 0, approaching a divergent profile in the *d*_2_ ≫ *d*_1_ limit (black curve). We consider an electron velocity *v* = 0.7 *c* and normalize the frequency and the probability using the smallest distance *d*_1_. **c** Decoherence probability as a function of *d*_2_/*d*_1_ for *d*_⊥_ = 0 and different electron velocities (see color scale)

Here, we theoretically demonstrate that the presence of an extended material structure can produce strong electron decoherence on electron beams (e-beams) placed at an arbitrarily large distance from the material. We consider radiative modes of commensurably large wavelengths, for which the materials behave either as real-permittivity dielectrics or lossless perfect electric conductors (PECs), such that material excitations can be ignored. Specifically, we consider a thin PEC half-plane and a two-path e-beam passing perpendicularly to it (Fig. 2a). Because the half-plane in the zero-thickness limit is a scale-invariant structure and the PEC response eliminates any absolute length scale from the problem at zero temperature, we find that the decoherence between the two paths only depends on the ratio of their distances to the half-plane, and consequently, decoherence is predicted to take place for arbitrarily large macroscopic electron–half-plane distances, provided the inter-path separation is sufficiently large. At finite vacuum temperature *T*, the thermal wavelength *λ*_*T*_ = 2*πℏc*/*k*_B_*T* plays a role by imposing an absolute length scale that is inversely proportional to *T* (e.g., *λ*_*T*_ ~ 14 mm at 1 K and 50 *μ*m at room temperature). We find that decoherence is then boosted for large inter-path separations compared with *λ*_*T*_, provided one of the paths passes near the half-plane. In a more practical scenario, we consider a finite-width ribbon and show that the half-plane limit is recovered for large width compared with the electron–edge distance. Our results support the use of electron decoherence to sense the presence of distant objects and measure the vacuum temperature.

## Results

### General theory of electron-beam decoherence

We are interested in investigating the loss of coherence among different spatial regions of a single electron prepared in a beam moving along *z* with velocity *v*. The electron state can change due to the interaction with the environment (i.e., any material structure and the radiation field), giving rise to inelastic components that are position-dependent and, thus, decreasing the degree of coherence between separate spatial regions of the beam. As a practical manifestation of this effect, after propagation from those regions to an electron detector, the loss of coherence produces a reduction in the visibility of the resulting interference fringes, which we investigate here in a rigorous quantitative manner.

Describing an incident electron through its interaction-picture density matrix ${\rho }_{e}^{i}({{{\bf{r}}}},{{{{\bf{r}}}}}^{{\prime} })$, scattering by a structure produces a final density matrix given by

1 $$\begin{array}{r}{\rho }_{e}^{f}({{{\bf{r}}}},{{{{\bf{r}}}}}^{{\prime} })={{{{\rm{e}}}}}^{-P({{{\bf{r}}}},{{{{\bf{r}}}}}^{{\prime} })+{{{\rm{i}}}}\chi ({{{\bf{r}}}},{{{{\bf{r}}}}}^{{\prime} })}{\rho }_{e}^{i}({{{\bf{r}}}},{{{{\bf{r}}}}}^{{\prime} })\end{array}$$

where

2 $$\begin{array}{ll}P({{{\bf{R}}}},{{{{\bf{R}}}}}^{{\prime} })\,=\,\displaystyle\frac{1}{2}\displaystyle\int_{0}^{\infty }d\omega \left[2{n}_{T}(\omega )+1\right]\\ \qquad\quad\quad\,\,\, \times \,\left[\Gamma ({{{\bf{R}}}},{{{\bf{R}}}},\omega )+\Gamma ({{{{\bf{R}}}}}^{{\prime} },{{{{\bf{R}}}}}^{{\prime} },\omega )-2\Gamma ({{{\bf{R}}}},{{{{\bf{R}}}}}^{{\prime} },\omega )\right]\end{array}$$

is the decoherence probability, which is in turn expressed as a frequency integral of the generalized loss probability

3 $$\begin{array}{rcl}\Gamma ({{{\bf{R}}}},{{{{\bf{R}}}}}^{{\prime} },\omega )&=&\displaystyle\frac{4{e}^{2}}{\hbar }\displaystyle\int_{-\infty }^{\infty }dz\displaystyle\int_{-\infty }^{\infty }d{z}^{{\prime} }\cos \left[\frac{\omega }{v}(z-{z}^{{\prime} })\right]\\ &\times &{{{\rm{Im}}}}\{-{G}_{zz}({{{\bf{r}}}},{{{{\bf{r}}}}}^{{\prime} },\omega )\}\end{array}$$

(a self-contained derivation of these expressions is presented in Supplementary Sections S1 and S2). Here, the temperature *T* enters through the Bose-Einstein distribution function ${n}_{T}(\omega )={\left({{{{\rm{e}}}}}^{\hbar \omega /{k}_{{{{\rm{B}}}}}T}-1\right)}^{-1}$, while the scattering structure is accounted for through the electromagnetic Green tensor $G({{{\bf{r}}}},{{{{\bf{r}}}}}^{{\prime} },\omega )$, which can be calculated by solving the macroscopic electromagnetic response according to $\nabla \times \nabla \times G({{{\bf{r}}}},{{{{\bf{r}}}}}^{{\prime} },\omega )-({\omega }^{2}/{c}^{2})\epsilon ({{{\bf{r}}}},\omega )G({{{\bf{r}}}},{{{{\bf{r}}}}}^{{\prime} },\omega )=(-1/{c}^{2})\delta ({{{\bf{r}}}}-{{{{\bf{r}}}}}^{{\prime} })$ for any structure defined by a local, frequency-dependent permittivity *ϵ*(**r**, *ω*). The real phase $\chi ({{{\bf{r}}}},{{{{\bf{r}}}}}^{{\prime} })$ in Eq. (1) is also expressed in terms of the Green function (see Supplementary Section S2), adding a rigid shift to the fringes observed in two-path interference. Following pioneering studies of decoherence in free-space electrons^23^, explicit results analogous to these expressions have been obtained by using macroscopic quantum electrodynamics^14,39^, but the derivation that we present in the SI is self-contained and formulated in more general terms. Incidentally, the decoherence probability *P* can take values larger than 1 since it must be understood as a depletion of coherence given by e^−*P*^ according to Eq. (1). Analogously, the classical EELS probability can also exceed unity and must be understood as the mean of a Poissonian distribution of multiple losses^47^.

### Electron decoherence by a half-plane

The application of Eqs. (2) and (3) to an e-beam passing outside and perpendicularly to a PEC half-plane produces analytical expressions for the decoherence probability, as shown in the self-contained derivation offered in the Supplementary Section S3. More precisely, referring to the geometry depicted in Fig. 2a, involving two paths with transverse coordinates **R**_1_ = (*d*_1_, 0) and **R**_2_ = (*d*_2_, *d*_⊥_), we find

4 $$\begin{array}{l}P({\bf{R}}_{1},{\bf{R}}_{2})=\displaystyle\frac{\alpha }{2\pi} \displaystyle \int_{0}^{1} \dfrac{d \mu}{\sqrt{1 - \mu^{2}}}\; \displaystyle \frac{\left[(1+v^{2}/c^{2}){\mu}^{2}+\eta^{2}\right]}{\left({\mu}^{2}+\eta^{2}\right)^{3/2}}\displaystyle\int_{0}^{\infty}\frac{d\theta }{\theta }\,\coth (\theta /4\pi )\\ \qquad\qquad\qquad \times \left[{\rm{e}}^{-2 \theta ({d}_{1}/{\lambda }_{T})\sqrt{{\mu}^{2}+{\eta}^{2}}}+{\rm{e}}^{-2\theta ({d}_{2}/{\lambda }_{T})\sqrt{{\mu}^{2}+{\eta}^{2}}}-2\cos \left({\mu} \theta \,{d}_{\perp }/{\lambda }_{T}\right)\,{\rm{e}}^{-\theta \left[({d}_{1}+{d}_{2})/{\lambda }_{T}\right]\sqrt{{\mu}^{2}+{\eta}^{2}}}\right]\end{array}$$

where *α* = *e*^2^/*ℏc* ≈ 1/137 is the fine structure constant and *η* = *c*/*vγ* is a velocity-dependent parameter that uses the relativistic Lorentz factor $\gamma =1/\sqrt{1-{v}^{2}/{c}^{2}}$. The integration variable *θ* = 2*πℏω*/*k*_B_*T* in Eq. (4) encapsulates the exchanged energy *ℏω*, and we have rewritten the thermal factor as

5 $$\begin{array}{r}2{n}_{T}(\omega )+1=\coth (\theta /4\pi )\end{array}$$

As we argue above, this factor and $\Gamma ({{{\bf{R}}}},{{{{\bf{R}}}}}^{{\prime} },\omega )$ are both diverging as 1/*ω* in the *ω* → 0 limit. However, the divergence is canceled because the expression inside square brackets in Eq. (4) behaves as ∝ *θ*^2^ ~ *ω*^2^ for small *ω*: the first two terms inside the square brackets represent the contributions arising from the two separate electron paths passing by **R**_1_ and **R**_2_, respectively, whereas the rightmost term stands for path interference, and while the integral of each of these three terms diverges, their sum is finite. Consequently, the decoherence probability *P*(**R**_1_, **R**_2_) remains finite. We note that this quantity vanishes for **R**_1_ = **R**_2_, as expected from Eq. (2), and it depends on **R**_1_, **R**_2_, and *T* only through the ratios *d*_1_/*λ*_*T*_, *d*_2_/*λ*_*T*_, and *d*_⊥_/*λ*_*T*_.

#### Zero-temperature limit

In the zero-temperature limit, we have *n*_*T*_(*ω*) → 0, so we can approximate $\coth (\theta /4\pi )\approx 1$ in Eq. (4) [see Eq. (5)]. The *θ* integral can then be performed analytically by first absorbing the $(1/{\lambda }_{T})\sqrt{{\mu }^{2}+{\eta }^{2}}$ factor of the exponentials into the integration variable, and then considering the identity $\int\nolimits_{0}^{\infty }d\theta \,{{{{\rm{e}}}}}^{-a\sqrt{{\theta }^{2}+{g}^{2}}}/\sqrt{{\theta }^{2}+{g}^{2}}={K}_{0}(ga)$ (see Eq. 3.914-4 in Ref. ^48^) together with the expansion ${K}_{0}(ga)=\log (2)-\log (ga)-{{{\mathcal{C}}}}+{{{\mathcal{O}}}}[{(ga)}^{2}\log (ga)]$ for *ga* ≪ 1, where ${{{\mathcal{C}}}}$ is the Euler constant. Applying this result to the three terms inside the square brackets of Eq. (4), setting *d*_⊥_ = 0, and taking the *g* → 0 limit, we find

6 $$\begin{array}{r}{P}_{T = 0}({d}_{1},{d}_{2},{d}_{\perp }=0)=\displaystyle\frac{\alpha }{2\pi }\,f(v/c)\,\log \left[\displaystyle\frac{{({d}_{1}+{d}_{2})}^{2}}{4{d}_{1}{d}_{2}}\right]\end{array}$$

where $f(v/c)=\int\nolimits_{0}^{1}d\mu \,\left[(1+v^2/c^2)\mu^2+\eta^2\right]\,(1-\mu^2)^{-1/2}\,{\left({\mu }^{2}+{\eta }^{2}\right)}^{-3/2}$ encapsulates the dependence on electron velocity via the variable *η* = *c*/*vγ*. Incidentally, this function admits the closed-form expression $f(\beta )=\beta\gamma\,\left(1+{\beta}^{2}\right)\,{\mathcal{K}}\left({-\beta}^{2}{\gamma}^{2}\right)-\left({\beta}^{3}/\gamma\right)\, {\mathcal{E}}\left({-\beta}^{2}{\gamma}^{2}\right)$ in terms of the elliptical integrals ${{{\mathcal{K}}}}$ and ${{{\mathcal{E}}}}$. Interestingly, the dependences on path positions and electron velocity are factorized in Eq. (6). The distance dependence of the decoherence probability exhibits a logarithmic divergence as $P({d}_{1},{d}_{2})\approx (\alpha /2\pi )\,f(v/c)\,| \log ({d}_{2}/{d}_{1})|$ in the *d*_2_/*d*_1_ → 0, *∞* limits, while it vanishes for *d*_1_ = *d*_2_. In addition, *P*(*d*_1_, *d*_2_) vanishes at *v* = 0 and diverges as $\propto \log \gamma$ as the electron velocity approaches the speed of light.

It is instructive to examine the frequency integral of Eq. (4) in the *T* = 0 limit. For *d*_⊥_ = 0 and *d*_2_ > *d*_1_, Fig. 2b shows that low frequencies become increasingly relevant as we increase *d*_2_, eventually converging to a profile that diverges as ∝ 1/*ω* at low frequencies in the *d*_2_/*d*_1_ → *∞* limit, for which the frequency integral is consequently infinite (i.e., we have full decoherence preventing any interference when mixing the two paths). We remark that an arbitrarily large loss of coherence can take place even when *d*_1_ is made arbitrarily large, provided *d*_2_/*d*_1_ ≫ 1, as the electron can always couple to long-wavelength excitations.

Universal curves for the decoherence probability are obtained from Eq. (6) for *d*_⊥_ = 0 as a function of *d*_2_/*d*_1_ (Fig. 2c) for different electron velocities. Despite the logarithmic divergence with *d*_2_/*d*_1_ and the $\propto \log \gamma$ divergence as *v* approaches *c*, the decoherence probability takes relatively small values at *T* = 0 within the wide range of distances and velocities explored in Fig. 2c. This conclusion is however dramatically changed at finite temperatures, as we show below.

Similar results as those presented in Fig. 2b, c are obtained for the zero-temperature decoherence probability when varying the inter-path distance *d*_⊥_ along the direction parallel to the half-plane edge while setting *d*_1_ = *d*_2_ (see Fig. S3 in SI), which we calculate by numerically integrating Eq. (4) after setting $\coth (\theta /4\pi )=1$.

#### Decoherence at finite temperature

**Fig. 3:**
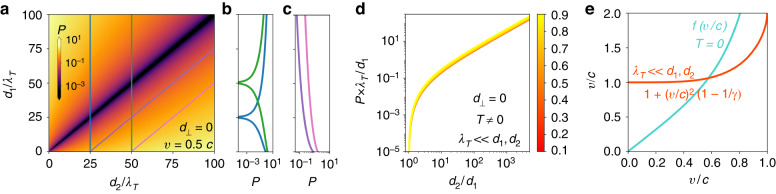
Decoherence at finite temperature. **a** Decoherence probability under the two-path configuration of Fig. 2a as a function of path-edge distances *d*_1_ and *d*_2_, normalized to the thermal wavelength *λ*_*T*_ = 2*πcℏ*/*k*_B_*T*. We set the electron velocity to *v* = 0.5 *c*. **b**, **c** Cuts of the probability in (**a**) along the color-coordinated lines. **d** Decoherence probability in the high-temperature limit (*d*_1_, *d*_2_ ≫ *λ*_*T*_), in which *λ*_*T*_*P*/*d*_1_ is only a function of *d*_2_/*d*_1_. **e** Electron-velocity prefactors in the *T* = 0 and *d*_1_, *d*_2_ ≫ *λ*_*T*_ limits. We set *d*_⊥_ = 0 in all cases

We examine the full dependence of the decoherence probability *P* [Eq. (4)] on *d*_1_/*λ*_*T*_ and *d*_2_/*λ*_*T*_ for *d*_⊥_ = 0 in Fig. 3a–c, setting *v*/*c* = 0.5 as an illustrative example since the dependence on velocity is relatively mild (see Fig. S4 in SI). The diagonal of the plot in Fig. 3a is dominated by a substantial reduction in the decoherence probability when ∣*d*_1_ − *d*_2_∣ ≲ *λ*_*T*_ (see also Fig. 3b; we note that *P* = 0 for *d*_1_ = *d*_2_). However, *P* quickly rises to large values when the distance difference is a few times the thermal wavelength (Fig. 3c).

It is interesting to analytically examine the high-temperature limit, in which the integral in Eq. (4) is dominated by regions where *n*_*T*_(*ω*) ≈ *k*_B_*T*/*ℏω* ≫ 1, so we can approximate $\coth (\theta /4\pi )\approx 4\pi /\theta$ [see Eq. (5)]. Setting again *d*_⊥_ = 0, and changing the *θ* variable of integration to absorb the $(1/{\lambda }_{T})\sqrt{{\mu }^{2}+{\eta }^{2}}$ factor, the *θ* integral can be analytically performed by using the identity $\int\nolimits_{0}^{\infty }d\theta \,{{{{\rm{e}}}}}^{-a\theta }/({\theta }^{2}+{g}^{2})=(1/g)[{{{\rm{Ci}}}}(ga)\sin (ga)-{{{\rm{Si}}}}(ga)\cos (ga)]$ (see Eq. 3.354-1 in Ref. ^48^), where Ci and Si are the cosine and sine integral functions, respectively. We then expand ${{{\rm{Ci}}}}(ga)={{{\mathcal{C}}}}+\log (ga)+{{{\mathcal{O}}}}{(ga)}^{2}$ and ${{{\rm{Si}}}}(ga)=-\pi /2+ga+{{{\mathcal{O}}}}{(ga)}^{2}$ for *ga* ≪ 1, and from here, we find $\int\nolimits_{0}^{\infty }d\theta \,{{{{\rm{e}}}}}^{-a\theta }/({\theta }^{2}+{g}^{2})\approx a\log a+\cdots \,$, where the eliminated terms are linear in *a*, independent of *a*, or vanishing in the *g* → 0 limit, so they do not contribute to the *θ* integral when summing the three exponential terms in Eq. (4). The *μ* integral of the remaining aloga contribution can also be performed in closed form, leading to the final result

7 $$\begin{array}{ll}{P}_{{d}_{1},{d}_{2}\gg {\lambda }_{T}}({d}_{1},{d}_{2},{d}_{\perp }=0)=2\pi\alpha \left[1+({v^2}/{c^2})\left(1-{1}/{\gamma}\right)\right]\\ \qquad\qquad\qquad\qquad\qquad\qquad \times \left[\displaystyle\frac{{d}_{1}}{{\lambda }_{T}}\log \left(\frac{2{d}_{1}}{{d}_{1}+{d}_{2}}\right)+\frac{{d}_{2}}{{\lambda }_{T}}\log \left(\frac{2{d}_{2}}{{d}_{1}+{d}_{2}}\right)\right]\end{array}$$

where we again observe a factorization of the dependences on electron–half-plane distances and electron velocity. The decoherence probability *P* exhibits a linear divergence with *d*_1_ and *d*_2_ for a constant ratio *d*_2_/*d*_1_ in the *d*_1_, *d*_2_ ≫ *λ*_*T*_ limit. In addition, the temperature enters through an overall factor 1/*λ*_*T*_ = *k*_B_*T*/2*πℏc* ∝ *T*, so that *P* also scales linearly with *T*.

In the high-temperature limit [Eq. (7)], the scaled probability *λ*_*T*_*P*/*d*_1_ only depends on the ratios *d*_2_/*d*_1_ and *v*/*c*, and in particular, it exhibits a roughly linear increase with *d*_2_/*d*_1_, as shown in Fig. 3d. We further observe the noted linear scaling with *T*, directly reflecting the linear increase with temperature in the photon population at long photon wavelengths (i.e., those that are commensurate with the electron–edge distances, which are large compared with *λ*_*T*_ in the limit under examination). In addition, the dependence on electron velocity is fully contained in the prefactor 1 ≤ 1 + (*v*^2^/*c*^2^)(1 − 1/γ) ≤ 2 in Eq. (7), which takes finite values over a broad range of velocities typically used in electron microscopes, down to *v* = 0 (Fig. 3e). This is in contrast to the *T* = 0 behavior, in which, although *P* also depends on velocity through a prefactor *f*(*v*/*c*) [see Eq. (6)], the latter vanishes in the small velocity limit and diverges when *v* approaches *c*.

We stress that the change in behavior from zero to finite temperate is continuous but relatively steep, as shown in Fig. S5 in SI.

### Finite-size effects: decoherence by a metallic ribbon

While the assumption of a thin PEC screen is reasonable for metallic films of small thickness compared with the electron–edge distances, a finite extension of the half-plane geometry can play a role because the aforementioned infrared divergence requires that the structure responds at arbitrarily low frequencies (see Table 1). We study finite-size effects by limiting the extension of the half-plane in one direction and considering instead a ribbon of finite width *W*. The decoherence probability is then computed by employing an ad hoc boundary-element method in which the ribbon is discretized through a uniform set of points along the transverse direction, as explained in the self-contained Supplementary Section S4.

**Fig. 4:**
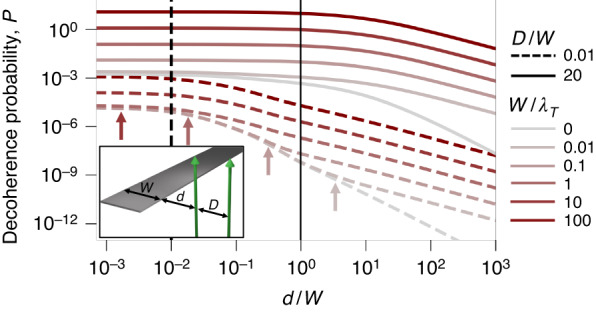
Finite size effects on the decoherence probability. We plot the decoherence probability *P* for the system sketched in the lower-left inset. The e-beam is prepared in a two-path superposition state (inter-path distance *D*) and interacts with a thin PEC ribbon of width *W*. The path-ribbon distances are *d* and *d* + *D*. We present results for different values of the *D*/*W* and *W*/*λ*_*T*_ ratios (see the legend on the right)

The resulting decoherence probability is plotted in Fig. 4 for a two-path configuration featuring an inter-path distance *D* and a shortest electron–ribbon distance *d* (see inset and Fig. S6 in SI). Specifically, we show calculations for *D*/*W* = 0.01 and 20, combined with different *W*/*λ*_*T*_ ratios ranging from zero temperature to *W* =100 *λ*_*T*_. At large inter-path separations (*D* = 20 *W*, solid curves), the infinite half-plane limit is recovered for distances *d* ≪ *W*. The condition *d* = *W* (vertical solid line) signals the transition between the half-plane limit and a regime in which the probability is exponentially attenuated when increasing *d* at all temperatures. This behavior is produced by ribbon-mediated coupling of the path that is closest to the edge to radiative modes, while the distant path experiences a negligible degree of inelastic interaction.

For relatively small inter-path separations (*D* = 0.01 *W*, dashed curves in Fig. 4), both paths undergo a similar level of inelastic interaction, and consequently, *P* is strongly reduced compared to the results for large *D*. Under these conditions, the half-plane limit is recovered at *d* ~ *D* = 0.01 *W* (vertical dashed line) in both the low- and high-temperature regimes (*W*/*λ*_*T*_ = 0 and ≫ 1, respectively). Similarly to the half-plane, a departure from the *T* = 0 regime is observed at large ribbon–electron separations, as indicated by color-coordinated vertical arrows in Fig. 4. At large temperatures, this departure takes place over the entire range of distances *d* considered in the figure, thus producing an overall increase in the decoherence probability.

## Discussion

In summary, inelastic radiative scattering of free electrons passing near extended structures produces a divergence in electron decoherence at high temperatures and/or large inter-path separations for electrons prepared in a two-path beam configuration. In essence, the extended material structure acts as a coupler between the evanescent electron field and long-wavelength free-space radiation. We exemplify this effect through an analytical treatment of the interaction between free electrons and a metallic half-plane, which is a self-scaling geometry such that, at zero temperature, there are no absolute length scales in the system, and therefore, the decoherence probability only depends on the ratio of electron-path distances to the edge. For this system, the probability that the electron interacts with radiative modes receives a divergent contribution at low frequencies and, although this yields an infinite frequency-integrated probability, the loss of coherence remains finite for finite electron inter-path separation. The decoherence probability increases with temperature as we depart from *T* = 0. Then, the thermal wavelength defines an absolute length scale in the system. These results require the involvement of low-frequency radiation, with wavelengths that are commensurate with both the electron-path–edge distances and the extension of the material, as confirmed by the observation that the half-plane limit is recovered when considering instead ribbons of large width compared with the thermal wavelength and the electron–edge distance.

These results suggest the possibility of detecting the presence of distant objects without perturbing them (i.e., without causing any inelastic excitation in the involved materials, and relying instead on decoherence produced by coupling to radiative modes). Indeed, at zero temperature, the self-scaling nature of the half-plane geometry implies that a large decoherence probability is obtained for any arbitrarily large electron–edge distance, provided the latter is small compared with the inter-path separation. In addition, at finite temperatures, a high degree of decoherence is observed when the inter-path distance is large compared with the thermal wavelength.

The strong temperature dependence of the decoherence probability could be exploited to perform vacuum thermometry and measure the temperature of the free-space thermal radiation bath. The required inter-path distances are a few times the thermal wavelength. At room temperature, the latter is *λ*_*T*_ ≈ 50 *μ*m, so we need to consider distances of hundreds of microns, which are typical separations between e-beams and different structural components in electron microscopes. Incidentally, some degree of undesired electron decoherence could be produced due to radiative coupling assisted by elements placed close to the e-beam in electron microscopes, an effect that deserves further examination in light of the results presented in this work.

Our predictions could therefore be tested in an electron microscope by introducing a specimen consisting of a wide ribbon (e.g., having width *W* = 20 *λ*_*T*_ ≈ 1 mm at room temperature) and splitting the e-beam into two paths (e.g., separated by a distance *D* = *W*). Then, interference between the two paths is significantly reduced when bringing the ribbon within a distance *d* < 0.1 *W* ≈ 0.1 mm from the nearest electron path (e.g., *P* > 0.2). We further conceive a macroscopic version of this experiment at cryogenic temperatures, for which the decoherence probability is preserved if all lengths are scaled by the thermal wavelength (e.g., multiplied by a factor of ~ 100 when moving from room temperature to outer-space thermal-background conditions at 2.7 K). The far-field interference arising when mixing electron paths that are separated by a distance *D* results in interference fringes with an angular spacing ∝ 1/*D*, which becomes too small to be experimentally resolved at macroscopic separations *D* of hundreds of microns. Instead, an electron optics system could be used to split an e-beam and separate the electron paths to the desired distance *D* in the region of interaction with the material structure, followed by a second set of optical components that bring the electron paths to interference at a post-selecting transmission grating^49,50^ (see Fig. S1 in SI).

The 1/*r* distance dependence of the Coulomb field that accompanies a moving charge (the electron) underscores the observed divergences in electron decoherence. An analogous divergence in decoherence could potentially be produced by other types of excitations that share similar long-range behavior. In particular, further investigation is needed to explore the effect of coupling between massive particle waves and gravitons, as well as the gravitational interaction with long-range modes in material structures (e.g., sound and elastic waves).

## Supplementary information

Supplemental Material

## Data Availability

All data needed to evaluate the conclusions in the paper are present in the paper and/or the Supplementary Materials.
