# Correction: Radiative loss of coherence in free electrons: a long-range quantum phenomenon

**DOI:** 10.1038/s41377-026-02376-5

**Published:** 2026-08-04

**Authors:** Cruz I. Velasco, Valerio Di Giulio, F. Javier García de Abajo

**Affiliations:** 1 ICFO-Institut de Ciencies Fotoniques, The Barcelona Institute of Science and Technology, 08860 Castelldefels (Barcelona), Spain; 2 ICREA-Institució Catalana de Recerca i Estudis Avançats, Passeig Lluís Companys 23, 08010 Barcelona, Spain

**Keywords:** Quantum optics, Quantum optics

Correction to: *Light: Science & Applications*10.1038/s41377-023-01361-6published online 26 January 2024

In the derivation of Sec. S3 of the Supplemental Material for the field induced by an electron passing near and perpendicular to a thin conducting half-plane, we identified an error in the ansatz of Eq. (S26a). The solution presented for the induced current ${{\bf{j}}}_{i}^{{\rm{ind}}}({{\bf{k}}}_{{\rm{||}}},\,\omega )$ leads to an unphysical divergence near the edge of the half-plane. This issue can be resolved by noting that the solution is not unique: there exists an alternative way of eliminating the branch cut in the upper *k*_*x*_ plane by moving the $\sqrt{K+{k}_{x}}$ factor from the numerator to the denominator in Eq. (S26a). By combining these two solutions (i.e., with $\sqrt{K+{k}_{x}}$ placed either in the numerator or in the denominator), multiplied by coefficients that depend on ω and *k*_*y*_, we obtain a unique form that avoids such pathological behavior in the induced current while still satisfying Eqs. (S24). Namely,

C1 $${{\bf{j}}}_{i}^{{\mathrm{ind}}}({k}_{\parallel },\omega )=\frac{{\rm{i}}\omega e}{v{K}^{2}}\frac{{{\rm{e}}}^{-(\kappa ,{\rm{i}}{k}_{y})\cdot {{\bf{R}}}_{i}}}{{k}_{x}+{\rm{i}}\kappa }\\\times\left[\frac{{K}^{2}\hat{{\bf{x}}}+{k}_{x}{k}_{y}\hat{{\bf{y}}}}{\sqrt{K+{\rm{i}}\kappa }\sqrt{K+{k}_{x}}}+\frac{{\rm{i}}{k}_{y}}{\kappa }\sqrt{K+{\rm{i}}\kappa }\sqrt{K+{k}_{x}}\hat{{\bf{y}}}\right]$$

This pathology-removal procedure follows a scheme similar to that used in previous analyses of the electromagnetic response of a thin metallic half-plane^1^, as also noted in the corrected Supplemental Material. This modification propagates to Eq. (S29) in the Supplemental Material, as well as to the equations immediately above and below it, which now should read

C2 $${\tilde{\Gamma}}({{\bf{R}}}_{1},{{\bf{R}}}_{2},\omega )=\frac{{e}^{2}}{\pi\hslash {c}^{2}}\displaystyle{\int_{\!0}^{k}}\frac{d{k}_{y}}{{\kappa }^{3}}\;\frac{{\kappa }^{2}+({c}^{2}/{v}^{2}){k}_{y}^{2}}{\sqrt{{k}^{2}-{k}_{y}^{2}}}\\\times\displaystyle{{\rm{e}}}^{-\kappa ({x}_{1}+{x}_{2})}\,{\rm{cos}}[{k}_{y}({y}_{1}-{y}_{2})]$$

and

C3 $$\Gamma ({{\bf{R}}}_{1},{{\bf{R}}}_{2},\omega )=\frac{\alpha }{\pi \omega }\displaystyle{\int _{0}^{1}}\frac{d\mu }{\sqrt{1-{\mu }^{2}}}\frac{[(1+{v}^{2}/{c}^{2}){\mu }^{2}+{\eta }^{2}]}{{({\mu }^{2}+{\eta }^{2})}^{{3/2}}}\\\times\displaystyle{\rm{cos}}[\mu \,k({y}_{1}-{y}_{2})]\,{{\rm{e}}}^{-k({x}_{1}+{x}_{2})\sqrt{{\mu }^{2}+{\eta }^{2}}}$$

for the generalized EELS probability, and

C4 $$P{({{\bf{R}}}_{1},{{\bf{R}}}_{2})=\frac{\alpha }{2\pi }{\int }_{0}^{1}\frac{d\mu }{\sqrt{1-{\mu }^{2}}}\,\frac{[(1+{v}^{2}/{c}^{2}){\mu }^{2}+{\eta }^{2}]}{{({\mu }^{2}+{\eta }^{2})}^{3/2}}{\int }_{0}^{\infty }\frac{d\theta }{\theta }\,\,{\rm{coth}}(\theta /4\pi)}\\\qquad\qquad\qquad{\times}\left[{{\rm{e}}}^{-2\theta ({d}_{1}/{\lambda }_{T})\sqrt{{\mu }^{2}+{\eta }^{2}}}+{{\rm{e}}}^{-2\theta ({d}_{2}/{\lambda }_{T})\sqrt{{\mu }^{2}+{\eta }^{2}}}\right.\\\qquad\qquad\qquad\left.-\,2\,{\rm{cos}}(\mu \theta \,{d}_{\perp }/{\lambda }_{T})\,{{\rm{e}}}^{-\theta [({d}_{1}+{d}_{2})/{\lambda }_{T}]\sqrt{{\mu }^{2}+{\eta }^{2}}}\right]$$

for the decoherence probability. Equation (4) in the main text must also be replaced by Eq. (C4).

From the zero-temperature limit of Eq. (C4), we also obtain a different result for the coefficient

C5 $$f(v/c)={\int }_{0}^{1}d\mu \,[(1+{v}^{2}/{c}^{2}){\mu }^{2}+{\eta }^{2}]\,{(1-{\mu }^{2})}^{-\mathrm{1/2}}\,{[{\mu }^{2}+{\eta }^{2}]}^{-\mathrm{3/2}}$$

in Eq. (4) of the main text, which admits the closed-form expression

C6 $$f(\beta )=\beta \gamma (1+{\beta }^{2})\,{\mathcal{K}}(-{\beta }^{2}{\gamma }^{2})-({\beta }^{3}/\gamma ) {\mathcal E} (-{\beta }^{2}{\gamma }^{2})$$

These expressions are now substituting the original ones right below Eq. (4) in the main text.

Likewise, in the high-temperature limit, Eq. (C4) reduces to

C7 $$\begin{array}{l}{P}_{{d}_{1},{d}_{2}\gg {\lambda }_{T}}({d}_{1},{d}_{2},{d}_{\perp }\mathrm{=0})\mathrm{=2}\pi \alpha [1+({v}^{2}/{c}^{2})(1-1/\gamma )]\\ \,\,\,\,\,\,\,\,\,\,\,\,\,\,\,\,\,\,\,\,\,\,\,\,\,\,\,\,\,\,\,\,\,\,\,\,\,\,\,\times \left[\frac{{d}_{1}}{{\lambda }_{T}}{\mathrm{log}}\left(\frac{2{d}_{1}}{{d}_{1}+{d}_{2}}\right)+\frac{{d}_{2}}{{\lambda }_{T}}{\mathrm{log}}\left(\frac{2{d}_{2}}{{d}_{1}+{d}_{2}}\right)\right]\end{array}\,$$

which substitutes Eq. (7) in the corrected main text, where we now specify that the dependence on electron velocity is fully contained in the prefactor $1\le 1+\left({v}^{2}/{c}^{2}\right)\left(1-1/\gamma \right)\le 2$.

The above changes in the analytical expressions lead to corresponding modifications in the figures. The qualitative behavior remains unchanged, although some numerical corrections are required. Below, we provide the corrected figures, indicating which ones they replace in the main text and in the Supplemental Material.

**CORRECTED FIGURES** 
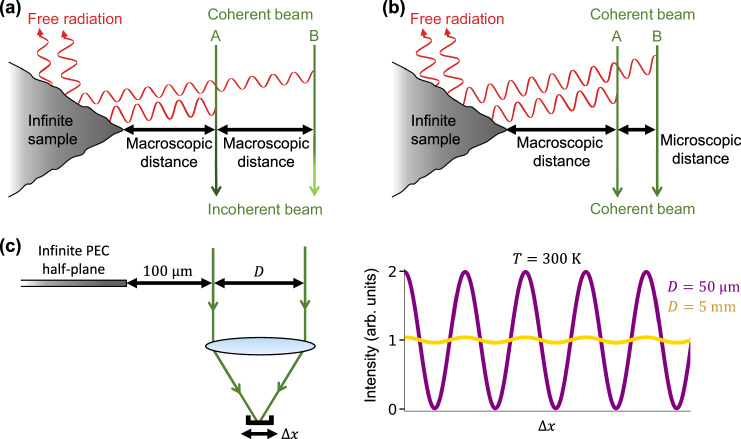


**FIG. C1**: Corrected version of Fig. 1 in the main text, featuring a slight change in the yellow curve in (**c**), which now oscillates with a smaller amplitude.
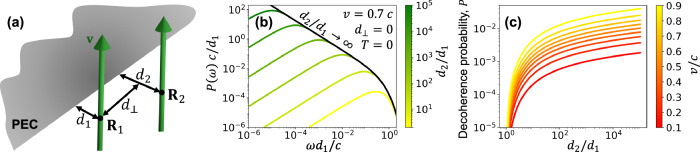


**FIG. C2:** Corrected version of Fig. 2 in the main text, showing an overall increase in *P* for the curves in (**b**) and (**c**).
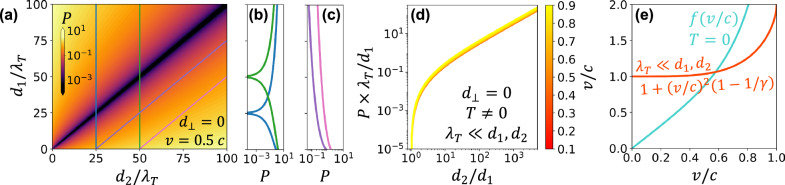


**FIG. C3:** Corrected version of Fig. 3 in the main text, showing an overall increase in P in (**a**)-(**d**) and in f(v/c) in (**e**). In (**a**)–(**c**), we have introduced a factor of α (the fine structure constant) that was missing in the original paper.
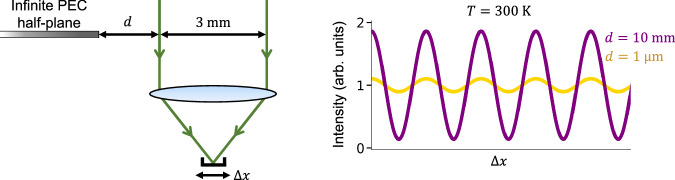


**FIG. C4:** Corrected version of Fig. S2 in the Supplemental Material, featuring a slight change in the yellow curve, which now oscillates with a smaller amplitude.
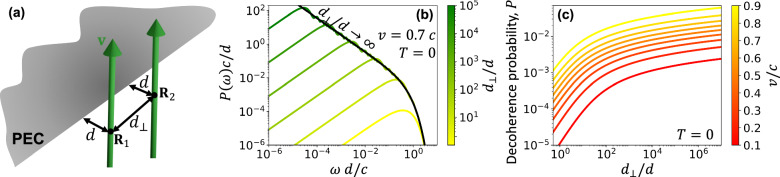


**FIG. C5:** Corrected version of Fig. S3 in the Supplemental Material, showing an overall increase in P.
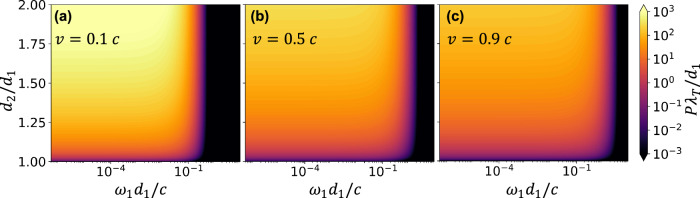


**FIG. C6:** Corrected version of Fig. S4 in the Supplemental Material, showing an overall increase in P.
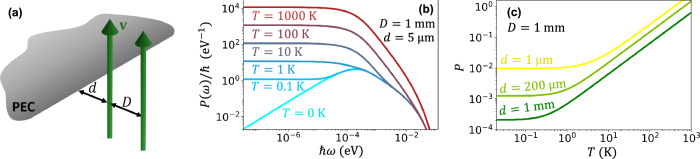


**FIG. C7:** Corrected version of Fig. S5 in the Supplemental Material, showing an overall increase in P.

## Supplementary information

Supplemental Material
