# Supplementary material for: Radiative loss of coherence in free electrons: a long-range quantum phenomenon
Source: Light Sci Appl. 2024 Jan 26;13:31. doi: 10.1038/s41377-023-01361-6 (PMC10810897; doi:10.1038/s41377-023-01361-6)
Supplement: Supplementary file 1 — Supplemental Material [file 41377_2023_1361_MOESM1_ESM.pdf]

Radiative loss of coherence in free electrons: a long-range quantum phenomenon

– SUPPLEMENTAL MATERIAL –

Cruz I. Velasco,[1] Valerio Di Giulio,[1] and F. Javier García de Abajo[1,2,*]

[1]*ICFO-Institut de Ciencies Fotoniques, The Barcelona Institute of Science and Technology, 08860 Castelldefels (Barcelona), Spain*

[2]*ICREA-Institució Catalana de Recerca i Estudis Avançats, Passeig Lluís Companys 23, 08010 Barcelona, Spain*

(Dated: March 27, 2026)

We present a self-contained derivation of the theory of electron decoherence based on first principles, along with supplementary results for a two-path electron beam oriented parallel to a half-plane edge, the spectral decomposition of the decoherence probability, the path-separation dependence of the decoherence probability produced by interaction with a ribbon, and a proposal for measuring electron interference fringes compatible with having a large inter-path separation at the position of the specimen.

Contents

S1. General description of electron-beam decoherence S1

S2. Decoherence by coupling to bosonic excitations S3

S3. Decoherence of an electron beam perpendicular to a perfectly conducting half-plane S5
- A. Electron-induced electric field S5
- B. Decoherence probability S6

S4. Decoherence by interaction with a metallic ribbon S7

S5. Low-frequency divergence of the EELS probability in extended structures S8

APPENDIX S8

A. Fluctuation-dissipation theorem for the vector potential S8

B. Derivation of Eq. (S14) S10

REFERENCES S10

SUPPLEMENTARY FIGURES S12

S1. GENERAL DESCRIPTION OF ELECTRON-BEAM DECOHERENCE

Starting from the Dirac equation to describe the electron and the radiation field, working in an electromagnetic gauge with zero scalar potential (the so-called temporal gauge), neglecting ponderomotive interactions, and adopting the nonrecoil approximation (i.e., considering an electron of high kinetic energy E_0 and constant electron velocity $\mathbf{v}$ under the assumption of small momentum exchanges with the environment compared with the initial electron momentum $\hbar\mathbf{q}_0$), one finds the approximate real-space Hamiltonian $\hat{\mathcal{H}}(\mathbf{r})=\hat{\mathcal{H}}_{\rm rad}+\hat{\mathcal{H}}_{\rm el}(\mathbf{r})+\hat{\mathcal{H}}_{\rm int}(\mathbf{r})$, where $\hat{\mathcal{H}}_{\rm rad}$ and $\hat{\mathcal{H}}_{\rm el}(\mathbf{r})=E_0-\hbar\mathbf{v}\cdot\mathbf{q}_0-{\rm i}\hbar\mathbf{v}\cdot\nabla$ describe the noninteracting radiation and electron components, while $\hat{\mathcal{H}}_{\rm int}(\mathbf{r})=(e\mathbf{v}/c)\cdot\hat{\mathbf{A}}(\mathbf{r})$ is the minimal-coupling radiation–electron interaction, which is simply proportional to the vector potential operator $\hat{\mathbf{A}}(\mathbf{r})$ (see Ref. [1] for a detailed derivation starting from the Dirac equation). It is useful to move to the interaction picture, in which the interaction Hamiltonian

---

*Electronic address: javier.garciadeabajo@nanophotonics.es

becomes

$$\hat{\mathcal{H}}_{\rm int}(\mathbf{r},t) = {\rm e}^{{\rm i}[\hat{\mathcal{H}}_{\rm rad}+\hat{\mathcal{H}}_{\rm el}(\mathbf{r})]t/\hbar}\,\hat{\mathcal{H}}_{\rm int}(\mathbf{r})\,{\rm e}^{-{\rm i}[\hat{\mathcal{H}}_{\rm rad}+\hat{\mathcal{H}}_{\rm el}(\mathbf{r})]t/\hbar}$$
$$= \frac{e\mathbf{v}}{c}\cdot\hat{\mathbf{A}}(\mathbf{r}+\mathbf{v}t,t), \tag{S1}$$

where $\hat{\mathbf{A}}(\mathbf{r})$ inside $\hat{\mathcal{H}}_{\rm int}(\mathbf{r})$ acquires a time dependence from both the transformation $\hat{\mathbf{A}}(\mathbf{r},t) = {\rm e}^{{\rm i}\hat{\mathcal{H}}_{\rm rad}t/\hbar}\hat{\mathbf{A}}(\mathbf{r}){\rm e}^{-{\rm i}\hat{\mathcal{H}}_{\rm rad}t/\hbar}$ and the displacement introduced by the $-{\rm i}\hbar\mathbf{v}\cdot\nabla$ term in $\hat{\mathcal{H}}_{\rm el}$ [i.e., we apply the identity [2] ${\rm e}^{\mathbf{v}t\cdot\nabla}f(\mathbf{r}){\rm e}^{-\mathbf{v}t\cdot\nabla}g(\mathbf{r}) = f(\mathbf{r}+\mathbf{v}t)g(\mathbf{r})$, which is valid for any $\mathbf{r}$-dependent functions $f(\mathbf{r})$ and $g(\mathbf{r})$].

It is convenient to describe the temporal dynamics through the evolution operator $\hat{\mathcal{S}}(\mathbf{r},t)$, defined in such a way that the combined electron–environment state at time t is given by $|\psi(\mathbf{r},t)\rangle = \hat{\mathcal{S}}(\mathbf{r},t)\,|\psi(\mathbf{r},-\infty)\rangle$, where $|\psi(\mathbf{r},-\infty)\rangle$ is the state in the infinite past and a ket notation is adopted to indicate the radiation degrees of freedom. To study the evolution of the electron, we propagate the full density matrix using $\hat{\mathcal{S}}(\mathbf{r},t)$ and eventually trace out the radiation. We consider the initial state of the electron–environment system to be uncorrelated [i.e., expressed as a tensor product of an electron density matrix $\rho_e^i(\mathbf{r},\mathbf{r}')$ and a thermal distribution of the environment degrees of freedom]. In addition, we neglect any memory effect in the environment, such that it can be approximated as a thermal state of constant temperature T at all times. Putting these elements together, the time-dependent electron-projected density matrix reads

$$\rho(\mathbf{r},\mathbf{r}',t) = \big\langle\hat{\mathcal{S}}(\mathbf{r},t)\hat{\mathcal{S}}^\dagger(\mathbf{r}',t)\big\rangle_T\,\rho_e^i(\mathbf{r},\mathbf{r}'), \tag{S2}$$

where $\langle\cdots\rangle_T$ indicates the thermal average over the radiation field. At any time after the interaction has taken place, this expression reduces to

$$\rho_e^f(\mathbf{r},\mathbf{r}') = {\rm e}^{-P(\mathbf{r},\mathbf{r}')+{\rm i}\chi(\mathbf{r},\mathbf{r}')}\rho_e^i(\mathbf{r},\mathbf{r}')$$

[Eq. (1) in the main text], where $P(\mathbf{r},\mathbf{r}')$ and $\chi(\mathbf{r},\mathbf{r}')$ are time-invariant quantitites that depend on z and z' only through the difference $z-z'$ for $\mathbf{v}$ along $\hat{\mathbf{z}}$ (see below). Note that, in the interaction picture, the density matrix $\rho(\mathbf{r},\mathbf{r}')$ does not evolve with time in the absence of any perturbation, while a trivial time dependence is acquired as $\rho(\mathbf{r}-\mathbf{v}t,\mathbf{r}'-\mathbf{v}t) = {\rm e}^{-{\rm i}\hat{\mathcal{H}}_{\rm el}(\mathbf{r})t/\hbar}\rho(\mathbf{r},\mathbf{r}'){\rm e}^{{\rm i}\hat{\mathcal{H}}_{\rm el}(\mathbf{r}')t/\hbar}$ in the Schrödinger picture, reflecting the translation due to the electron motion, which is computed again through the aforementioned displacement. This allows us to write

$$\rho(\mathbf{r}-\mathbf{v}t,\mathbf{r}'-\mathbf{v}t) = {\rm e}^{-P(\mathbf{r},\mathbf{r}')+{\rm i}\chi(\mathbf{r},\mathbf{r}')}\rho_e^i(\mathbf{r}-\mathbf{v}t,\mathbf{r}'-\mathbf{v}t) \tag{S3}$$

for the electron density matrix in the Schrödinger picture. Here, the real functions $P(\mathbf{r},\mathbf{r}')$ and $\chi(\mathbf{r},\mathbf{r}')$ are defined by

$${\rm e}^{-P(\mathbf{r},\mathbf{r}')+{\rm i}\chi(\mathbf{r},\mathbf{r}')} = \big\langle\hat{\mathcal{S}}(\mathbf{r},\infty)\hat{\mathcal{S}}^\dagger(\mathbf{r}',\infty)\big\rangle_T, \tag{S4}$$

such that they account for spatially dependent decoherence and an elastic phase [3], respectively. To find more explicit expressions for $P(\mathbf{r},\mathbf{r}')$ and $\chi(\mathbf{r},\mathbf{r}')$, we use the Magnus expansion up to second order [4] and approximate the evolution operator as

$$\hat{\mathcal{S}}(\mathbf{r},t) \approx \exp\bigg\{-\frac{\rm i}{\hbar}\int_{-\infty}^{t}dt'\,\hat{\mathcal{H}}_{\rm int}(\mathbf{r},t') \tag{S5}$$
$$-\frac{1}{2\hbar^2}\int_{-\infty}^{t}dt'\int_{-\infty}^{t'}dt''\,\big[\hat{\mathcal{H}}_{\rm int}(\mathbf{r},t'),\hat{\mathcal{H}}_{\rm int}(\mathbf{r},t'')\big]\bigg\}.$$

Now, introducing Eq. (S1) into Eq. (S5), we can write $\hat{\mathcal{S}}(\mathbf{r},\infty) = \exp\{-\hat{N}_1(\mathbf{r})-(1/2)\hat{N}_2(\mathbf{r})\}$ in terms of the operators

$$\hat{N}_1(\mathbf{r}) = \frac{{\rm i}ev}{\hbar c}\int_{-\infty}^{\infty}dt\,\hat{A}_z(\mathbf{r}-\mathbf{v}t,t), \tag{S6a}$$

$$\hat{N}_2(\mathbf{r}) = \Big(\frac{ev}{\hbar c}\Big)^2\int_{-\infty}^{\infty}dt\int_{-\infty}^{t}dt'\,\big[\hat{A}_z(\mathbf{r}-\mathbf{v}t,t),\hat{A}_z(\mathbf{r}-\mathbf{v}t',t')\big], \tag{S6b}$$

where we set $\mathbf{v} = v\,\hat{\mathbf{z}}$ without loss of generality. Then, using the Baker-Campbell-Hausdorff formula and retaining only terms up to second order in $\hat{\mathbf{A}}$, Eq. (S4) becomes

$${\rm e}^{-P(\mathbf{r},\mathbf{r}')+{\rm i}\chi(\mathbf{r},\mathbf{r}')} \approx \bigg\langle\exp\Big(-\hat{N}_1(\mathbf{r})+\hat{N}_1(\mathbf{r}')-\frac{1}{2}\big\{[\hat{N}_1(\mathbf{r}),\hat{N}_1(\mathbf{r}')]+\hat{N}_2(\mathbf{r})-\hat{N}_2(\mathbf{r}')\big\}\Big)\bigg\rangle_T, \tag{S7}$$

where we have employed the properties $\hat{N}_1^{\dagger} = -\hat{N}_1$ and $\hat{N}_2^{\dagger} = -\hat{N}_2$. Finally, we use the cumulant expansion $\langle e^{\lambda\hat{C}}\rangle_T = \exp\big(\sum_{m=1}^{\infty}\lambda^m C_m\big)$ to evaluate Eq. (S7) up to second order in $\hat{\mathbf{A}}$, for which one needs to Taylor-expand both sides of this expression around $\lambda = 0$, identify terms proportional to each power of the variable λ, and finally set $\lambda = 1$. Following this procedure, we readily find $C_1 = \langle\hat{C}\rangle_T$ and $C_2 = (1/2)\big(\langle\hat{C}^2\rangle_T - \langle\hat{C}\rangle_T^2\big)$. Setting $\hat{C} = -\hat{N}_1(\mathbf{r}) + \hat{N}_1(\mathbf{r}') - (1/2)\big\{[\hat{N}_1(\mathbf{r}), \hat{N}_1(\mathbf{r}')] + \hat{N}_2(\mathbf{r}) - \hat{N}_2(\mathbf{r}')\big\}$ in accordance with Eq. (S7), we finally obtain

$$\begin{aligned}-P(\mathbf{r},\mathbf{r}') + \mathrm{i}\chi(\mathbf{r},\mathbf{r}') \approx \frac{1}{2}\Big\langle \big[\hat{N}_1(\mathbf{r}) - \hat{N}_1(\mathbf{r}')\big]^2 - \big[\hat{N}_1(\mathbf{r}), \hat{N}_1(\mathbf{r}')\big] - \hat{N}_2(\mathbf{r}) + \hat{N}_2(\mathbf{r}')\Big\rangle_T \\ - \big\langle \hat{N}_1(\mathbf{r}) - \hat{N}_1(\mathbf{r}')\big\rangle_T - \frac{1}{2}\big\langle \hat{N}_1(\mathbf{r}) - \hat{N}_1(\mathbf{r}')\big\rangle_T^2 \end{aligned} \tag{S8}$$

to second order in the interaction. Equations (S6) and (S8) can be applied to any vector potential incorporating the optical response of a material structure through the radiation modes in $\hat{\mathbf{A}}(\mathbf{r}, t)$. Importantly, Eqs. (S5), (S7), and (S8) become exact in a commonly encountered scenario when $\hat{\mathbf{A}}(\mathbf{r}, t)$ is constructed from bosonic operators (see Sec. S2). Nevertheless, Eq. (S8) remains valid to second order even if the radiation modes involve coupling to non-bosonic excitations, such as those resulting from scattering by systems with a finite number of energy levels or when nonlinear response effects are significant.

S2. DECOHERENCE BY COUPLING TO BOSONIC EXCITATIONS

In most practical situations, the radiation field is composed of bosonic modes representing free photons as well as polarization and scattering by material structures. Then, each mode j contributes with a term $\alpha_j^*\hat{a}_j^{\dagger} + \alpha_j\hat{a}_j$ to the vector potential operator $\hat{\mathbf{A}}_z(\mathbf{r}, t)$, where $\hat{a}_j^{\dagger}$ and $\hat{a}_j$ are creation and annihilation operators, while α_j are spatiotemporally dependent complex coefficients. The bosonic commutation relations $[\hat{a}_j, \hat{a}_{j'}^{\dagger}] = \delta_{jj'}$ directly imply that $[\hat{\mathcal{H}}_{\rm int}(\mathbf{r}, t), \hat{\mathcal{H}}_{\rm int}(\mathbf{r}', t')]$ is a c-number, and consequently, we have $[\hat{\mathcal{H}}_{\rm int}(\mathbf{r}, t), [\hat{\mathcal{H}}_{\rm int}(\mathbf{r}', t'), \hat{\mathcal{H}}_{\rm int}(\mathbf{r}'', t'')]] = 0$. This result renders Eqs. (S5) and (S7) exact, as all terms beyond second order (which we neglected in the Magnus expansion and the Baker-Campbell-Hausdorff formula) are proportional to commutators of three of more interaction terms.

Likewise, when computing the thermal average in Eq. (S7), we note that $\hat{N}_2(\mathbf{r})$ [Eq. (S6b)] and $[\hat{N}_1(\mathbf{r}), \hat{N}_1(\mathbf{r}')]$ [see Eq. (S6a)] are both commutators of vector potential operators and, therefore, c-numbers that can be pulled outside $\langle\dots\rangle_T$. In addition, $\hat{N}_1(\mathbf{r}) - \hat{N}_1(\mathbf{r}')$ becomes a sum over terms of the form $\beta_j^*\hat{a}_j^{\dagger} - \beta_j\hat{a}_j$, where β_j are complex coefficients that depend on $\mathbf{r}$ and $\mathbf{r}'$. Using the identity [5, 6] $\big\langle e^{c_j^*\hat{a}_j^{\dagger} - c_j\hat{a}_j}\big\rangle_T = \exp\big\{(1/2)\big\langle(c_j^*\hat{a}_j^{\dagger} - c_j\hat{a}_j)^2\big\rangle_T\big\}$ together with $\big\langle\big[\sum_j(c_j^*\hat{a}_j^{\dagger} - c_j\hat{a}_j)\big]^2\big\rangle_T = \big\langle\sum_j(c_j^*\hat{a}_j^{\dagger} - c_j\hat{a}_j)^2\big\rangle_T$ (i.e., cross terms vanish under the thermal average), we then obtain $\big\langle e^{-\hat{N}_1(\mathbf{r}) + \hat{N}_1(\mathbf{r}')}\big\rangle_T = \exp\big\{(1/2)\big\langle[\hat{N}_1(\mathbf{r}) - \hat{N}_1(\mathbf{r}')]^2\big\rangle_T\big\}$. From these considerations, we find that Eq. (S7) leads to the exact relation (for bosonic excitations)

$$-P(\mathbf{r},\mathbf{r}') + \mathrm{i}\chi(\mathbf{r},\mathbf{r}') = \frac{1}{2}\Big[\Big\langle\big[\hat{N}_1(\mathbf{r}) - \hat{N}_1(\mathbf{r}')\big]^2\Big\rangle_T - \big[\hat{N}_1(\mathbf{r}), \hat{N}_1(\mathbf{r}')\big] - \hat{N}_2(\mathbf{r}) + \hat{N}_2(\mathbf{r}')\Big], \tag{S9}$$

with $\hat{N}_1(\mathbf{r})$ and $\hat{N}_2(\mathbf{r})$ defined in Eqs. (S6). This expression coincides with Eq. (S8) because $\langle\hat{N}_1(\mathbf{r})\rangle_T = 0$ for these types of modes.

To evaluate Eq. (S9), we use the relation

$$\langle\hat{A}_a(\mathbf{r}, t)\hat{A}_{a'}(\mathbf{r}', t')\rangle_T = -4\hbar c^2\int_0^{\infty} d\omega\, \mathrm{Im}\{G_{aa'}(\mathbf{r}, \mathbf{r}', \omega)\}\,\Big\{2n_T(\omega)\cos[\omega(t - t')] + e^{-\mathrm{i}\omega(t-t')}\Big\} \tag{S10}$$

for the thermal average of the product of two vector potential operators, which is self-consistently derived in Appendix A. Here, a and a' denote Cartesian components, we introduce the Bose-Einstein distribution function

$$n_T(\omega) = \frac{1}{e^{\hbar\omega/k_{\rm B}T} - 1} \tag{S11}$$

at temperature T, and $G(\mathbf{r}, \mathbf{r}', \omega)$ is the electromagnetic Green tensor, implicitly defined by the equation

$$\nabla\times\nabla\times G(\mathbf{r}, \mathbf{r}', \omega) - \frac{\omega^2}{c^2}\epsilon(\mathbf{r}, \omega)\, G(\mathbf{r}, \mathbf{r}', \omega) = -\frac{1}{c^2}\delta(\mathbf{r} - \mathbf{r}'), \tag{S12}$$

where $\epsilon(\mathbf{r},\omega)$ is the space- and frequency-dependent local permittivity. In practice, $G(\mathbf{r},\mathbf{r}',\omega)$ can be calculated analytically in simple geometries (e.g., spherical and planar interfaces) or via numerical electromagnetic simulations. From Eq. (S10), using the reciprocity property $G_{aa'}(\mathbf{r},\mathbf{r}',\omega)=G_{a'a}(\mathbf{r}',\mathbf{r},\omega)$, we readily find

$$[\hat{A}_a(\mathbf{r},t),\hat{A}_{a'}(\mathbf{r}',t')]=8\mathrm{i}\hbar c^2\int_0^\infty d\omega\ \sin\big[\omega(t-t')\big]\ \mathrm{Im}\big\{G_{aa'}(\mathbf{r},\mathbf{r}',\omega)\big\}, \tag{S13}$$

where we have dismissed $\langle\cdots\rangle_T$ because the commutator is a c-number for bosonic operators (see above) and, therefore, independent of the state of the radiation field.

Using Eq. (S13), we can transform Eq. (S6b) into

$$\hat{N}_2(\mathbf{R})=\frac{4e^2\,\mathrm{i}}{\hbar}\int_{-\infty}^\infty dz\int_{-\infty}^\infty dz'\int_0^\infty d\omega\ \cos\Big[\frac{\omega}{v}(z-z')\Big]\ \mathrm{Re}\big\{G_{zz}(\mathbf{R},z,\mathbf{R},z',\omega)\big\} \tag{S14}$$

(see Appendix B), where we have indicated that the result only depends on the transverse coordinates $\mathbf{R}=(x,y)$. Likewise, Eq. (S10) allows us to directly evaluate the N_1 terms in Eq. (S9) and finally write

$$\begin{aligned}\chi(\mathbf{r},\mathbf{r}')=\frac{2e^2}{\hbar}\int_{-\infty}^\infty dz''\int_{-\infty}^\infty dz'''\int_0^\infty d\omega\ \bigg\{&2\sin\Big[\frac{\omega}{v}(z-z'-z''+z''')\Big]\ \mathrm{Im}\big\{G_{zz}(\mathbf{R},z'',\mathbf{R}',z''',\omega)\big\}\\&+\cos\Big[\frac{\omega}{v}(z''-z''')\Big]\ \mathrm{Re}\big\{G_{zz}(\mathbf{R}',z'',\mathbf{R}',z''',\omega)-G_{zz}(\mathbf{R},z'',\mathbf{R},z''',\omega)\big\}\bigg\},\end{aligned} \tag{S15a}$$

$$P(\mathbf{r},\mathbf{r}')=\frac{1}{2}\int_0^\infty d\omega\ \big[2n_T(\omega)+1\big]\ \big[\Gamma(\mathbf{r},\mathbf{r},\omega)+\Gamma(\mathbf{r}',\mathbf{r}',\omega)-2\,\Gamma(\mathbf{r},\mathbf{r}',\omega)\big], \tag{S15b}$$

where

$$\Gamma(\mathbf{r},\mathbf{r}',\omega)=\frac{4e^2}{\hbar}\int_{-\infty}^\infty dz''\int_{-\infty}^\infty dz'''\ \cos\Big[\frac{\omega}{v}(z-z'-z''+z''')\Big]\ \mathrm{Im}\big\{-G_{zz}(\mathbf{R},z'',\mathbf{R}',z''',\omega)\big\}. \tag{S16}$$

The decoherence probability in Eqs. (S15b) and (S16) coincides with the result derived in Ref. [7]. For $z=z'$, these expressions reduce to

$$P(\mathbf{R},\mathbf{R}')=\frac{1}{2}\int_0^\infty d\omega\big[2n_T(\omega)+1\big]\ \Big[\Gamma(\mathbf{R},\mathbf{R},\omega)+\Gamma(\mathbf{R}',\mathbf{R}',\omega)-2\Gamma(\mathbf{R},\mathbf{R}',\omega)\Big]$$

$$\Gamma(\mathbf{R},\mathbf{R}',\omega)=\frac{4e^2}{\hbar}\int_{-\infty}^\infty dz\int_{-\infty}^\infty dz'\cos\Big[\frac{\omega}{v}(z-z')\Big]\ \mathrm{Im}\big\{-G_{zz}(\mathbf{r},\mathbf{r}',\omega)\big\}$$

[Eqs. (2) and (3) in the main text]. The present analysis remains valid to any order of interaction for quasi-monochromatic electrons in general under the nonrecoil approximation, thus generalizing previous calculations for a classical electron [3] and for a quantum electron within second-order perturbation theory [8]. Reassuringly, these functions satisfy the properties $P(\mathbf{r},\mathbf{r})=\chi(\mathbf{r},\mathbf{r})=0$ (thus guaranteeing the conservation of the total electron probability), $P(\mathbf{r},\mathbf{r}')=P(\mathbf{r}',\mathbf{r})$, and $\chi(\mathbf{r},\mathbf{r}')=-\chi(\mathbf{r}',\mathbf{r})$ (i.e., reciprocity is inherited from the Green tensor). Incidentally, the latter (symmetry with respect to the exchange of $\mathbf{r}$ and $\mathbf{r}'$) could already be anticipated by applying the Hermiticity of the density matrix $\rho(\mathbf{r},\mathbf{r}')=\rho^*(\mathbf{r}',\mathbf{r})$ to Eq. (S3). The present self-contained derivation relies just on the assumption of bosonic excitations and the nonrecoil approximation. We note that macroscopic quantum electrodynamics [9] provides an alternative framework to derive Eqs. (S15).

We note that Eq. (S16), which relates the position-dependent electron decoherence to the electromagnetic Green tensor in the structure under consideration, bears some similarity to the electron energy-loss probability (EELS) [10]. Within the linear and nonrecoil approximations, the latter can be derived by separately considering each frequency component of the external current $\mathbf{j}(\mathbf{r},\omega)=-e\hat{\mathbf{z}}\,\mathrm{e}^{\mathrm{i}\omega z/v}\,\delta(\mathbf{R}-\mathbf{R}')$ associated with a classical electron beam passing by the transverse position $\mathbf{R}'$. The electric field generated by this current at a position $\mathbf{r}$ is then obtained by applying the Green tensor as defined in Eq. (S12), yielding

$$E_z(\mathbf{r},\mathbf{R}',\omega)=4\pi\mathrm{i}\,e\,\omega\int_{-\infty}^\infty dz'\ G_{zz}(\mathbf{r},\mathbf{r}',\omega)\ \mathrm{e}^{\mathrm{i}\omega z'/v}. \tag{S17}$$

Following the methods in Ref. [10], we find

$$\begin{aligned}\tilde{\Gamma}(\mathbf{R},\mathbf{R}',\omega)&=\frac{e}{\pi\hbar\omega}\int_{-\infty}^\infty dz\ \mathrm{Re}\big\{\mathrm{e}^{-\mathrm{i}\omega z/v}\,E_z(\mathbf{r},\mathbf{R}',\omega)\big\}\\&=\frac{4e^2}{\hbar}\int_{-\infty}^\infty dz\int_{-\infty}^\infty dz'\ \mathrm{Im}\big\{-\mathrm{e}^{-\mathrm{i}\omega(z-z')/v}\,G_{zz}(\mathbf{r},\mathbf{r}',\omega)\big\}\end{aligned} \tag{S18}$$

for a generalized EELS probability associated with a probe electron passing by the transverse position $\mathbf{R}$. In particular, $\Gamma(\mathbf{R},\mathbf{R},\omega)$ is the regular EELS probability. Finally, Eq. (S16) can be recast as

$$\Gamma(\mathbf{R},\mathbf{R}',\omega)=\frac{1}{2}\big[\tilde{\Gamma}(\mathbf{R}',\mathbf{R},\omega)+\tilde{\Gamma}(\mathbf{R},\mathbf{R}',\omega)\big] \tag{S19}$$

when considering positions $z=z'$ (i.e., within the same plane in an interferometric measurement). In practice, we can then evaluate $P(\mathbf{R},\mathbf{R}')$ in Eq. (S15b) [with $z=z'$, so the result is independent of z and z' according to Eq. (S16)] by obtaining $\Gamma(\mathbf{R},\mathbf{R}',\omega)$ from Eqs. (S18) and (S19) using the frequency-space classical electric field $E_z(\mathbf{r},\mathbf{R}',\omega)$ [Eq. (S17)] produced at $\mathbf{r}$ by an electron moving with velocity $\mathbf{v}=v\,\hat{\mathbf{z}}$ and transverse coordinates $\mathbf{R}'$. It is useful to note that only the induced part of the electric field $\mathbf{E}^{\rm ind}$ makes a contribution because the direct field, related to the free-space Green tensor $G^0(\mathbf{r},\mathbf{r}',\omega)$ [i.e., separating $G(\mathbf{r},\mathbf{r}',\omega)=G^0(\mathbf{r},\mathbf{r}',\omega)+G^{\rm ind}(\mathbf{r},\mathbf{r}',\omega)$], only contains components proportional to $\mathrm{e}^{\mathrm{i}k_z z'}$ with a z-projected wave vector $|k_z|\le\omega/c$ (i.e., within the light cone), thus vanishing after integration in Eq. (S17).

S3. DECOHERENCE OF AN ELECTRON BEAM PERPENDICULAR TO A PERFECTLY CONDUCTING HALF-PLANE

We apply the formalism developed in Sec. S2 to an electron beam moving perpendicularly to a perfectly conducting metallic half-plane and prepared in a superposition of two paths defined by the transverse coordinates $\mathbf{R}_i$ with $i=1,2$ (see Fig. 2a in the main text). The half-plane is taken to occupy the $x<0$ region of the $z=0$ plane and the electron is moving with velocity v along z without intersecting the metal (i.e., x_1, $x_2>0$). We proceed by first calculating the induced electric field component $E_z^{\rm ind}(\mathbf{r},z,\mathbf{R}_i,\omega)$ produced by each of the paths i [see Eq. (S17)], from which the decoherence probability $P(\mathbf{R},\mathbf{R}')$ (for $z=z'$) is computed by using Eq. (S15b) with $\Gamma(\mathbf{R}_i,z,\mathbf{R}_{i'},\omega)$ given by Eqs. (S18) and (S19). As we argue at the end of Sec. S2, the direct electric field produces a vanishing contribution to the decoherence probability, and therefore, we only calculate the field induced by the presence of the half-plane. It should also be noted that the present calculation can directly be applied to a laterally extended beam, but we retain the two-path terminology for clarity.

A. Electron-induced electric field

From the well-known expression for the electric field produced by an external source in free space [11], the two-dimensional current $\mathbf{j}_i^{\rm ind}(\mathbf{R},\omega)$ (i.e., $\perp\hat{\mathbf{z}}$) induced on the half-plane by the electron path passing by $\mathbf{R}_i$ gives

$$\mathbf{E}^{\rm ind}(\mathbf{r},\mathbf{R}_i,\omega)=\frac{\mathrm{i}}{\omega}\big(k^2\mathcal{I}_3+\nabla\otimes\nabla\big)\cdot\int d^2\mathbf{R}'\,\frac{\mathrm{e}^{\mathrm{i}k|\mathbf{r}-\mathbf{R}'|}}{|\mathbf{r}-\mathbf{R}'|}\,\mathbf{j}_i^{\rm ind}(\mathbf{R}',\omega), \tag{S20}$$

where $k=\omega/c$ is the light wavenumber, $\mathcal{I}_n$ denotes the $n\times n$ identity matrix, and we indicate the dependence on $\mathbf{R}_i$ by adopting the notation introduced in Eq. (S17). Using the identity

$$\frac{\mathrm{e}^{\mathrm{i}k|\mathbf{r}-\mathbf{R}'|}}{|\mathbf{r}-\mathbf{R}'|}=\frac{\mathrm{i}}{2\pi}\int\frac{d^2\mathbf{k}_\parallel}{k_z}\,\mathrm{e}^{\mathrm{i}\mathbf{k}_\parallel\cdot(\mathbf{R}-\mathbf{R}')+\mathrm{i}k_z|z|}, \tag{S21}$$

with $\mathbf{k}_\parallel=(k_x,k_y)$, $k_z=\sqrt{k^2-k_\parallel^2+\mathrm{i}0^+}$, and $\mathrm{Im}\{k_z\}>0$, we can work out Eq. (S20) to write the in-plane induced electric field as

$$\mathbf{E}_\parallel^{\rm ind}(\mathbf{R},z=0,\mathbf{R}_i,\omega)=-\frac{1}{2\pi\omega}\int\frac{d^2\mathbf{k}_\parallel}{k_z}\,\mathrm{e}^{\mathrm{i}\mathbf{k}_\parallel\cdot\mathbf{R}}\mathcal{M}(\mathbf{k}_\parallel,\omega)\cdot\mathbf{j}_i^{\rm ind}(\mathbf{k}_\parallel,\omega), \tag{S22}$$

where $\mathbf{j}_i^{\rm ind}(\mathbf{k}_\parallel,\omega)=\int d^2\mathbf{R}'\,\mathrm{e}^{-\mathrm{i}\mathbf{k}_\parallel\cdot\mathbf{R}}\,\mathbf{j}_i^{\rm ind}(\mathbf{R},\omega)$ is the induced current in momentum space and we define the 2×2 matrix $\mathcal{M}(\mathbf{k}_\parallel,\omega)=k^2\mathcal{I}_2-\mathbf{k}_\parallel\otimes\mathbf{k}_\parallel$.

In the limit of a perfect conductor, both $\mathbf{E}_\parallel$ and the normal magnetic field H_z must vanish on the surface of the half-plane. From Faraday's law, the vanishing of $\mathbf{E}_\parallel$ directly implies $H_z=(\mathrm{i}/k)\hat{\mathbf{z}}\cdot(\nabla\times\mathbf{E}_\parallel)=(\mathrm{i}/k)(\hat{\mathbf{z}}\times\nabla_\parallel)\cdot\mathbf{E}_\parallel=0$, so we only need to consider the electric field. We then have $\mathbf{E}_\parallel^{\rm ind}(\mathbf{R},z=0,\mathbf{R}_i,\omega)=-\mathbf{E}_\parallel^{\rm ext}(\mathbf{R},z=0,\mathbf{R}_i,\omega)$, where [10]

$$\begin{aligned}\mathbf{E}_\parallel^{\rm ext}(\mathbf{R},z=0,\mathbf{R}_i,\omega)&=\frac{\mathrm{i}e}{\pi v}\int d^2\mathbf{k}_\parallel\,\frac{\mathbf{k}_\parallel\,\mathrm{e}^{\mathrm{i}\mathbf{k}_\parallel\cdot(\mathbf{R}-\mathbf{R}_i)}}{k_\parallel^2+(\omega/v\gamma)^2}\\&=\frac{e}{v}\int_{-\infty}^{\infty}dk_y\,\mathrm{e}^{(\kappa,\mathrm{i}k_y)\cdot(\mathbf{R}-\mathbf{R}_i)}\,\big[\hat{\mathbf{x}}+(\mathrm{i}k_y/\kappa)\,\hat{\mathbf{y}}\big]\end{aligned} \tag{S23}$$

is the in-plane external field due to the electron path passing by $\mathbf{R}_i$, $\gamma = 1/\sqrt{1-v^2/c^2}$ is the relativistic Lorentz factor, and we define

$$\kappa = \sqrt{(\omega/v\gamma)^2 + k_y^2}.$$

In Eq. (S23), we obtain the rightmost expression by closing the integration contour over the lower complex k_x plane (because we evaluate the field at positions $x < 0 < x_i$ in the half-plane), where only the pole $k_x = -\mathrm{i}\kappa$ contributes. Combining Eqs. (S22) and (S23), and working in (x, k_y, ω) space, the vanishing of the in-plane electric field at the half-plane leads to the condition

$$\int_{-\infty}^{\infty} \frac{dk_x}{k_z}\, \mathrm{e}^{\mathrm{i}k_x x} \mathcal{M}(\mathbf{k}_\parallel, \omega) \cdot \mathbf{j}_i^{\mathrm{ind}}(\mathbf{k}_\parallel, \omega) = \mathbf{e}_i(k_y, \omega)\, \mathrm{e}^{\kappa x}, \tag{S24a}$$

for $x < 0$, where

$$\mathbf{e}_i(k_y, \omega) = \frac{2\pi\omega e}{v}\, \mathrm{e}^{-(\kappa, \mathrm{i}k_y)\cdot\mathbf{R}_i} \left[\hat{\mathbf{x}} + (\mathrm{i}k_y/\kappa)\,\hat{\mathbf{y}}\right].$$

This needs to be supplemented by the condition of zero current outside the half-plane ($x > 0$):

$$\int_{-\infty}^{\infty} dk_x\, \mathrm{e}^{\mathrm{i}k_x x}\, \mathbf{j}_i^{\mathrm{ind}}(\mathbf{k}_\parallel, \omega) = 0. \tag{S24b}$$

The system formed by Eqs. (S24) determines a unique solution for the current. Following the methods in Ref. [12], we write

$$k_z = \sqrt{K - k_x}\sqrt{K + k_x} \tag{S25}$$

with $K = \sqrt{k^2 - k_y^2 + \mathrm{i}0^+}$ defined with the square root yielding a positive imaginary part. Noticing that the right-hand side of Eq. (S24a) depends on x just through a factor $\mathrm{e}^{\kappa x}$, we can anticipate the solution

$$\mathbf{j}_i^{\mathrm{ind}}(\mathbf{k}_\parallel, \omega) = \frac{\mathrm{i}\omega e}{vK^2}\, \frac{\mathrm{e}^{-(\kappa, \mathrm{i}k_y)\cdot\mathbf{R}_i}}{k_x + \mathrm{i}\kappa} \left[\frac{K^2\hat{\mathbf{x}} + k_x k_y \hat{\mathbf{y}}}{\sqrt{K + \mathrm{i}\kappa}\sqrt{K + k_x}} + \frac{\mathrm{i}k_y}{\kappa}\, \sqrt{K + \mathrm{i}\kappa}\sqrt{K + k_x}\, \hat{\mathbf{y}}\right], \tag{S26}$$

where the prefactor to the left of the square brackets incorporates a pole at $k_x = -\mathrm{i}\kappa$, which gives rise to the aforementioned x dependence upon integration of Eq. (S24a). This expression is the superposition of the two possible solutions that eliminate the branch cut in the upper k_x plane by either multiplying or dividing by $\sqrt{K + k_x}$, without introducing additional poles. The ω- and k_y-dependent coefficients weighting these two solutions are determined by enforcing Eq. (S24a), together with the condition that the electromagnetic energy remains finite despite the edge divergence [13], implying that the x and y components of the current must decay at least as $k_x^{-3/2}$ and $k_x^{-1/2}$, respectively, in the large $|k_x|$ limit. To verify that Eq. (S26) is indeed a solution of Eq. (S24b), we note that the integrand vanishes as $\mathrm{Im}\{k_x\} \to \infty$, so we can close the integration contour over the upper complex k_x plane. Although $\mathbf{j}_i^{\mathrm{ind}}(\mathbf{k}_\parallel, \omega)$ has one pole ($k_x = -\mathrm{i}\kappa$) and one branch cut ($k_x = -K$), they are both lying on the lower plane, and consequently, the integral is indeed zero. Likewise, the integrand in Eq. (S24a) vanishes as $\mathrm{Im}\{k_x\} \to -\infty$ and has a single branch cut [14] ($k_x = K$), which lies on the upper complex k_x plane, so we close the integration contour over the lower plane, yielding the right-hand side of the equation via the $k_x = -\mathrm{i}\kappa$ pole.

To study the interaction between the two electron paths, we need to consider the z component of the field induced at $x > 0$ [see Eq. (S18)]. From Eq. (S20), using Eq. (S21) again, we find

$$E_z^{\mathrm{ind}}(\mathbf{r}, \mathbf{R}_i, \omega) = \frac{\mathrm{sign}\{z\}}{2\pi\omega} \int d^2\mathbf{k}_\parallel\, \mathrm{e}^{\mathrm{i}(\mathbf{k}_\parallel\cdot\mathbf{R} + k_z|z|)}\, \mathbf{k}_\parallel \cdot \mathbf{j}_i^{\mathrm{ind}}(\mathbf{k}_\parallel, \omega), \tag{S27}$$

where $\mathbf{j}_i^{\mathrm{ind}}(\mathbf{k}_\parallel, \omega)$ is explicitly given by Eq. (S26).

B. Decoherence probability

We obtain the generalized EELS probability by inserting Eq. (S27) into Eq. (S18) and carrying out the z integral as

$$\int_{-\infty}^{\infty} dz\, \mathrm{e}^{\mathrm{i}k_z|z| - \mathrm{i}\omega z/v}\, \mathrm{sign}\{z\} = -\frac{2\mathrm{i}\omega/v}{k_x^2 + \kappa^2}. \tag{S28}$$

Noticing that $x_i > 0$ at the path positions $i = 1, 2$, we can perform the k_x integral by closing the contour over the upper complex plane, where $\mathbf{j}_i^{\rm ind}(\mathbf{k}_\parallel, \omega)$ is free from poles and branch cuts, while the result in Eq. (S28) contributes with a $k_x = {\rm i}\kappa$ pole, yielding

$$\tilde{\Gamma}(\mathbf{R}_1, \mathbf{R}_2, \omega) = \frac{e^2}{\pi\hbar c^2} \int_0^k \frac{dk_y}{\kappa^3} \frac{\kappa^2 + (c^2/v^2)k_y^2}{\sqrt{k^2 - k_y^2}} \, {\rm e}^{-\kappa(x_1+x_2)} \cos\left[k_y(y_1 - y_2)\right].$$

In this particular instance, we find $\tilde{\Gamma}(\mathbf{R}_1, \mathbf{R}_2, \omega) = \tilde{\Gamma}(\mathbf{R}_2, \mathbf{R}_1, \omega)$, so Eq. (S19) becomes

$$\Gamma(\mathbf{R}_1, \mathbf{R}_2, \omega) = \frac{\alpha}{\pi\omega} \int_0^1 \frac{d\mu}{\sqrt{1-\mu^2}} \frac{\left[(1 + v^2/c^2)\mu^2 + \eta^2\right]}{\left(\mu^2 + \eta^2\right)^{3/2}} \cos\left[\mu \, k(y_1 - y_2)\right] {\rm e}^{-k(x_1+x_2)\sqrt{\mu^2+\eta^2}}, \tag{S29}$$

where $\alpha = e^2/\hbar c \approx 1/137$ is the fine structure constant, $\eta = c/v\gamma$ is a velocity-dependent parameter, and $\mu = k_y/k$. Finally, we insert Eq. (S29) into Eq. (S15b) to obtain the decoherence probability

$$P(\mathbf{R}_1, \mathbf{R}_2) = \frac{\alpha}{2\pi} \int_0^1 \frac{d\mu}{\sqrt{1-\mu^2}} \frac{\left[(1 + v^2/c^2)\mu^2 + \eta^2\right]}{\left(\mu^2 + \eta^2\right)^{3/2}} \int_0^\infty \frac{d\theta}{\theta} \coth(\theta/4\pi)$$
$$\times \left[{\rm e}^{-2\theta\,(d_1/\lambda_T)\sqrt{\mu^2+\eta^2}} + {\rm e}^{-2\theta\,(d_2/\lambda_T)\sqrt{\mu^2+\eta^2}} - 2\cos\left(\mu\theta\, d_\perp/\lambda_T\right) {\rm e}^{-\theta\,[(d_1+d_2)/\lambda_T]\sqrt{\mu^2+\eta^2}}\right]$$

[Eq. (4) in the main text], where we have made the changes of variables $\theta = 2\pi\hbar\omega/k_{\rm B}T$ and $\mu = k_y/k$.

S4. DECOHERENCE BY INTERACTION WITH A METALLIC RIBBON

To investigate the role played by the size of the structure, we consider a zero-thickness, perfectly conducting ribbon of width W lying on the $z = 0$ plane, defined by edges at $x = 0$ and $x = -W$, and having infinite extension along y. We start from Eq. (S24a), now restricted to the $-W < x < 0$ region (the ribbon) and write $\mathbf{j}_i^{\rm ind}(\mathbf{k}_\parallel, \omega) = \int_{-W}^0 dx' \, e^{-{\rm i}k_x x'} \, \mathbf{j}_i^{\rm ind}(x', k_y, \omega)$ in terms of the x-space current $\mathbf{j}_i^{\rm ind}(x, k_y, \omega)$ (also limited to the ribbon area). After inserting this expression into Eq. (S24a), we carry out the k_x integral by using the identity $\int_{-\infty}^\infty dk_x \, {\rm e}^{{\rm i}k_x(x-x')} \left(k^2 - k_y^2 - k_x^2 + {\rm i}0^+\right)^{-1/2} = 2{\rm i}K_0\big(Q\,|x - x'|\big)$ (see Eq. 3.754-2 in Ref. [15]), where we define

$$Q = \sqrt{k_y^2 - k^2 - {\rm i}0^+}$$

with the square root taken to yield a positive real part. This leads to

$$2{\rm i} \int_{-W}^0 dx' \left[k^2 \mathcal{I}_2 + (\partial_x, {\rm i}k_y) \otimes (\partial_x, {\rm i}k_y)\right] \cdot \mathbf{j}_i^{\rm ind}(x', k_y, \omega) \, K_0\big(Q\,|x - x'|\big) = \mathbf{e}_i(k_y, \omega)\, {\rm e}^{\kappa x} \tag{S30}$$

with x in the $(-W, 0)$ range. Incidentally, this expression can also be obtained starting from the induced field in Eq. (S20) after Fourier-transforming in y.

We find $\mathbf{j}_i^{\rm ind}(x, k_y, \omega)$ numerically from Eq. (S30) by discretizing the integral through a set of N equally spaced points $\tilde{x}_j = -W + (j + 1/2)W/N$ labeled by $j = 0, \cdots, N-1$, each of them representing an interval $\tilde{x}_j - h/2 < x < \tilde{x}_j + h/2$ of width $h = W/N$. By approximating

$$\mathbf{j}_i^{\rm ind}(x, k_y, \omega) \approx \mathbf{j}_i^{\rm ind}(\tilde{x}_j, k_y, \omega) \equiv (2\pi\omega e/v)\, {\rm e}^{-(\kappa, {\rm i}k_y)\cdot\mathbf{R}_i} \, \mathbf{J}_j(k_y, \omega) \tag{S31}$$

(i.e., constant within each interval j), we transform Eq. (S30) into the $N \times N$ linear system

$$\sum_{j'=0}^{N-1} \left[\left(k^2\mathcal{I}_2 - k_y^2\,\hat{\mathbf{y}}\otimes\hat{\mathbf{y}}\right) I_{jj'}^0 + {\rm i}k_y\left(\hat{\mathbf{x}}\otimes\hat{\mathbf{y}} + \hat{\mathbf{y}}\otimes\hat{\mathbf{x}}\right) I_{jj'}^1 + \hat{\mathbf{x}}\otimes\hat{\mathbf{x}}\, I_{jj'}^2\right] \cdot \mathbf{J}_{j'}(k_y, \omega) = \left[\hat{\mathbf{x}} + ({\rm i}k_y/\kappa)\,\hat{\mathbf{y}}\right] {\rm e}^{\kappa\tilde{x}_j},$$

where $I_{jj'}^n = H^n(\tilde{x}_j - \tilde{x}_{j'} + h/2) - H^n(\tilde{x}_j - \tilde{x}_{j'} - h/2)$ are interval integrals defined in terms of the functions

$$H^0(x) = 2{\rm i} \int dx\, K_0\big(Q\,|x|\big) = -{\rm i}\,\pi\, x\left[\mathbf{L}_{-1}\big(Q\,|x|\big)\, K_0\big(Q\,|x|\big) + \mathbf{L}_0\big(Q\,|x|\big)\, K_1\big(Q\,|x|\big)\right] + C,$$
$$H^1(x) = 2{\rm i} \int dx\, \partial_x K_0\big(Q\,|x|\big) = -2\,{\rm i}\, K_0\big(Q\,|x|\big) + C,$$
$$H^2(x) = 2{\rm i} \int dx\, \partial_{xx} K_0\big(Q\,|x|\big) = 2\,{\rm i}\, {\rm sig}\{x\}\, Q\, K_1\big(Q\,|x|\big) + C,$$

with $\mathbf{L}_n$ denoting modified Struve functions.

From the induced current, we then combine Eqs. (S18), (S27), and (S28) to obtain

$$\tilde{\Gamma}(\mathbf{R}_1,\mathbf{R}_2,\omega)=\frac{e}{\pi\hbar\omega v}\int_{-\infty}^{\infty}dk_y\int_{-W}^{0}dx\,\mathrm{e}^{\kappa x}\,\mathrm{Re}\Big\{\mathrm{e}^{(-\kappa,\mathrm{i}k_y)\cdot\mathbf{R}_1}\,\big[\hat{\mathbf{x}}-\mathrm{i}(k_y/\kappa)\hat{\mathbf{y}}\big]\cdot\mathbf{j}_2^{\mathrm{ind}}(x,k_y,\omega)\Big\}.$$

Then, using the discretized solution [see Eq. (S31)] and applying Eq. (S19), we find

$$\Gamma(\mathbf{R}_1,\mathbf{R}_2,\omega)=\frac{4\alpha c}{v^2}\int_{-\infty}^{\infty}\frac{dk_y}{\kappa}\,\sinh(\kappa h/2)\,\mathrm{e}^{-\kappa(x_1+x_2)}\,\cos\big[k_y(y_1-y_2)\big]\,\mathrm{Re}\Big\{\big[\hat{\mathbf{x}}-\mathrm{i}(k_y/\kappa)\hat{\mathbf{y}}\big]\cdot\sum_{j=0}^{N-1}\mathrm{e}^{\kappa\tilde{x}_j}\mathbf{J}_j(k_y,\omega)\Big\}.$$

Finally, we compute the decoherence probability by inserting this expression into Eq. (S15b).

S5. LOW-FREQUENCY DIVERGENCE OF THE EELS PROBABILITY IN EXTENDED STRUCTURES

In the introduction of the main text, we argue that the frequency-resolved EELS probability $\Gamma(\omega)$ (i.e., a function of energy loss $\hbar\omega$) vanishes in the low-frequency limit for finite objects, but it diverges for extended structures. At zero temperature, the EELS probability is given by Eq. (S16) with $\mathbf{r}=\mathbf{r}'$. For a finite particle and small ω, this expression is proportional to $\mathrm{Im}\{\alpha(\omega)\}$ [8], where $\alpha(\omega)$ is the polarizability. In the presence of inelastic absorption, we have $\mathrm{Im}\{\alpha(\omega)\}\propto\omega$, while in the absence of absorption the optical theorem [16] (i.e., $\mathrm{Im}\{\alpha^{-1}(\omega)\}=2k^3/3$) prescribes a probability $\propto\omega^3$ due to radiative losses. This result can be intuitively understood from the increasingly small size of the particle compared to the light wavelength as ω is reduced. In addition, at finite temperature T, the inelastic scattering probability associated with the coupling to bosonic modes (e.g., photons and polaritons) must be multiplied by a factor $n_T(\omega)+1$ for electron energy losses ($\omega>0$) and $n_T(-\omega)$ for gains ($\omega<0$) [8], where the Bose-Einstein distribution function $n_T(\omega)$ [Eq. (S11)] introduces an additional factor $\propto 1/\omega$ at low frequencies. Consequently, the low-ω scaling of the loss probability for finite structures at finite temperatures is $\propto\omega^0$ in the presence of inelastic absorption and $\propto\omega^2$ for lossless objects. These conclusions are summarized in Table 1 in the main text (left columns).

For extended structures (e.g., a half-plane), the EELS probability was shown to diverge as $\propto 1/\omega$ at zero temperature due to radiative coupling [17]. The divergence becomes more dramatic (as $\propto 1/\omega^2$) at finite temperature due to the additional factor $n_T(\omega)+1$ for energy losses and $n_T(\omega)$ for gains. These conclusions are summarized in Table 1 in the main text (right column). Reassuringly, these divergences do not produce any unphysical results, and in particular, the frequency-integrated electron energy change [i.e., $\Delta E=\int_{-\infty}^{\infty}d\omega\,\hbar\omega\,\Gamma(\omega)$] is temperature-independent because the $n_T(\omega)$ terms cancel when adding losses and gains. Furthermore, ΔE is finite because the remaining $\propto 1/\omega$ divergence is suppressed when multiplied by the energy loss $\hbar\omega$. We also note that the decoherence probability studied above involves a similar cancelation among $\Gamma(\mathbf{R},\mathbf{R}',\omega)$ terms in Eq. (2) of the main text.

APPENDIX

Appendix A: Fluctuation-dissipation theorem for the vector potential

We intend to derive Eqs. (S10) and (S13), which are essentially the fluctuation-dissipation theorem [18, 19] for the vector potential operator $\hat{\mathbf{A}}(\mathbf{r},t)$. Our starting point is an interaction Hamiltonian

$$\hat{\mathcal{H}}_{\mathrm{int}}(t)=-\frac{1}{c}\int d^3\mathbf{r}\,\mathbf{j}(\mathbf{r},t)\cdot\hat{\mathbf{A}}(\mathbf{r},t)$$

in the interaction picture within the so-called temporal gauge (i.e., with the scalar potential set to zero), with $\mathbf{j}(\mathbf{r},t)$ representing a classical external current. We also consider a complete set of states $|n\rangle$ of the free radiation Hamiltonian $\hat{\mathcal{H}}_{\mathrm{rad}}$, satisfying $\hat{\mathcal{H}}_{\mathrm{rad}}\,|n\rangle=\hbar\omega_n\,|n\rangle$. Applying first-order perturbation theory under the assumption of a vanishing interaction in the infinite past [i.e., $\hat{\mathcal{H}}_{\mathrm{int}}(-\infty)=0$], the time-dependent perturbed states become

$$|\psi_n(t)\rangle\approx|n\rangle-\frac{\mathrm{i}}{\hbar}\int_{-\infty}^{t}dt'\,\hat{\mathcal{H}}_{\mathrm{int}}(t')\,|n\rangle\,.$$

We now introduce a thermal average at temperature T, so the expected value of the induced vector potential reduces to

$$A_a(\mathbf{r},t)=\langle\hat{A}_a(\mathbf{r},t)\rangle_T=\frac{1}{Z}\sum_n \mathrm{e}^{-\hbar\omega_n/k_\mathrm{B}T}\left[\,\langle\psi_n(t)|\,\hat{A}_a(\mathbf{r},t)\,|\psi_n(t)\rangle-\langle n|\,\hat{A}_a(\mathbf{r},t)\,|n\rangle\,\right]$$
$$\approx\frac{\mathrm{i}}{\hbar c\,Z}\sum_{n,a'}\mathrm{e}^{-\hbar\omega_n/k_\mathrm{B}T}\int_{-\infty}^{t}dt'\int d^3\mathbf{r}'\,\langle n|\left[\hat{A}_a(\mathbf{r},t),\hat{A}_{a'}(\mathbf{r}',t')\right]|n\rangle\;j_{a'}(\mathbf{r}',t')$$
$$=-4\pi c\int d^3\mathbf{r}'\int_{-\infty}^{\infty}dt'\sum_{a'}G_{aa'}(\mathbf{r},\mathbf{r}',t-t')\,j_{a'}(\mathbf{r}',t')$$

to first order in the external current. Here, $Z=\sum_n \mathrm{e}^{-\hbar\omega_n/k_\mathrm{B}T}$ is the partition function,

$$G_{aa'}(\mathbf{r},\mathbf{r}',t-t')=-\frac{\mathrm{i}}{4\pi\hbar c^2\,Z}\Theta(t-t')\,\sum_n \mathrm{e}^{-\hbar\omega_n/k_\mathrm{B}T}\,\langle n|\left[\hat{A}_a(\mathbf{r},t),\hat{A}_{a'}(\mathbf{r}',t')\right]|n\rangle \tag{A1}$$

is the electromagnetic Green tensor, and we use indices a and a' to label Cartesian components. The Green tensor must satisfy Eq. (S12) to guarantee that the average potential $\mathbf{A}(\mathbf{r},t)$ fulfills the macroscopic Maxwell equations in the presence of a source current $\mathbf{j}(\mathbf{r},t)$ [20]. We move to the frequency domain by Fourier-transforming the Green tensor as

$$G_{aa'}(\mathbf{r},\mathbf{r}',\omega)=\int_{-\infty}^{\infty}d\tau\;G_{aa'}(\mathbf{r},\mathbf{r}',\tau)\,\mathrm{e}^{\mathrm{i}\omega\tau}$$
$$=\frac{1}{4\pi\hbar c^2\,Z}\sum_{nn'}\langle n|\,\hat{A}_a(\mathbf{r})\,|n'\rangle\,\langle n'|\,\hat{A}_{a'}(\mathbf{r}')\,|n\rangle\;\frac{\mathrm{e}^{-\hbar\omega_n/k_\mathrm{B}T}-\mathrm{e}^{-\hbar\omega_{n'}/k_\mathrm{B}T}}{\omega+\omega_n-\omega_{n'}+\mathrm{i}0^+}$$
$$=\frac{1}{4\pi\hbar c^2}\int_{-\infty}^{\infty}\frac{d\omega'}{\omega-\omega'+\mathrm{i}0^+}\;\frac{S_{aa'}(\mathbf{r},\mathbf{r}',\omega')}{n_T(\omega')+1}, \tag{A2}$$

where the second line is obtained by inserting the unity operator $\sum_n |n\rangle\langle n|$ in the $\hat{A}\hat{A}$ products of Eq. (A1) and further writing $\hat{\mathbf{A}}(\mathbf{r},t)=\mathrm{e}^{\mathrm{i}\hat{\mathcal{H}}_\mathrm{rad}t/\hbar}\hat{\mathbf{A}}(\mathbf{r})\mathrm{e}^{-\mathrm{i}\hat{\mathcal{H}}_\mathrm{rad}t/\hbar}$ in terms of the vector potential operator $\hat{\mathbf{A}}(\mathbf{r})$ in the Schrödinger picture. Then, the third line is expressed in terms of the Bose-Einstein distribution function $n_T(\omega)$ [Eq. (S11)] and we define the spectral tensor

$$S_{aa'}(\mathbf{r},\mathbf{r}',\omega)=\frac{1}{Z}\sum_{nn'}\mathrm{e}^{-\hbar\omega_n/k_BT}\;\langle n|\,\hat{A}_a(\mathbf{r})\,|n'\rangle\,\langle n'|\,\hat{A}_{a'}(\mathbf{r}')\,|n\rangle\;\delta(\omega+\omega_n-\omega_{n'}).$$

By construction, the Green tensor in Eq. (A2) trivially satisfies the causality property

$$G(\mathbf{r},\mathbf{r}',\omega)=G^*(\mathbf{r},\mathbf{r}',-\omega), \tag{A3a}$$

and its poles are all lying on the lower complex ω plane. In addition, the spectral tensor is real for systems with time-reversal symmetry [21], implying that the reciprocity property $S_{aa'}(\mathbf{r},\mathbf{r}',\omega)=S_{a'a}(\mathbf{r}',\mathbf{r},\omega)$ is fulfilled and further transferred to the Green tensor:

$$G_{aa'}(\mathbf{r},\mathbf{r}',\omega)=G_{a'a}(\mathbf{r}',\mathbf{r},\omega). \tag{A3b}$$

Since the spectral tensor is real, we can write it as

$$S_{aa'}(\mathbf{r},\mathbf{r}',\omega)=-4\hbar c^2\,\left[n_T(\omega)+1\right]\,\mathrm{Im}\big\{G_{aa'}(\mathbf{r},\mathbf{r}',\omega)\big\} \tag{A4}$$

directly from Eq. (A2). We note that a temperature dependence is introduced in the Green tensor through the thermal average in Eq. (A1), which we dismiss in this work because it is small unless high temperatures affecting the properties of the involved materials are considered.

We follow an analogous procedure to calculate the correlations of the vector potential operator in the frequency domain, $\hat{\mathbf{A}}(\mathbf{r},\omega)=\int dt\;\mathrm{e}^{\mathrm{i}\omega t}\,\hat{\mathbf{A}}(\mathbf{r},t)$. Using the unity operator $\sum_n |n\rangle\langle n|$ and expressing the potentials in the Schrödinger picture, one finds

$$\big\langle\hat{A}_a(\mathbf{r},\omega)\hat{A}_{a'}(\mathbf{r}',\omega')\big\rangle_T=\frac{1}{Z}\int_{-\infty}^{\infty}dt\int_{-\infty}^{\infty}dt'\;\mathrm{e}^{\mathrm{i}\omega t+\mathrm{i}\omega' t'}\sum_{nn'}\mathrm{e}^{-\hbar\omega_n/k_BT}\;\mathrm{e}^{i(\omega_n-\omega_{n'})(t-t')}\,\langle n|\,\hat{A}_a(\mathbf{r})\,|n'\rangle\,\langle n'|\,\hat{A}_{a'}(\mathbf{r}')\,|n\rangle$$
$$=4\pi^2\;S_{aa'}(\mathbf{r},\mathbf{r}',\omega)\;\delta(\omega+\omega'),$$

which, together with Eq. (A4), leads to

$$\big\langle\hat{A}_a(\mathbf{r},\omega)\hat{A}_{a'}(\mathbf{r}',\omega')\big\rangle_T=-16\pi^2\,\hbar c^2\;\delta(\omega+\omega')\;\left[n_T(\omega)+1\right]\,\mathrm{Im}\big\{G_{aa'}(\mathbf{r},\mathbf{r}',\omega)\big\}. \tag{A5}$$

Finally, performing the inverse Fourier transform of Eq. (A5) (in both ω and ω') and using causality [Eq. (A3a)] together with the identity $n_T(\omega)+1=-n_T(-\omega)$, we readily obtain Eq. (S10).

Now, exchanging the order of the $\hat{A}$ operators in Eq. (A5) and applying reciprocity and causality [Eqs. (A3)], we obtain

$$\big\langle \hat{A}_{a'}(\mathbf{r}',\omega')\hat{A}_a(\mathbf{r},\omega)\big\rangle_T = -16\pi^2\,\hbar c^2\,\delta(\omega+\omega')\,n_T(\omega)\,\mathrm{Im}\big\{G_{aa'}(\mathbf{r},\mathbf{r}',\omega)\big\},$$

which, combined with Eq. (A5), produces the commutator

$$\big\langle[\hat{A}_a(\mathbf{r},\omega),\hat{A}_{a'}(\mathbf{r}',\omega')]\big\rangle_T = -16\pi^2\,\hbar c^2\,\delta(\omega+\omega')\,\mathrm{Im}\big\{G_{aa'}(\mathbf{r},\mathbf{r}',\omega)\big\},$$

whose inverse Fourier transform directly yields Eq. (S13).

Appendix B: Derivation of Eq. (S14)

We use the commutator in Eq. (S13) to transform Eq. (S6b) into

$$\hat{N}_2(\mathbf{r}) = \mathrm{i}\,\frac{8e^2v^2}{\hbar}\int_{-\infty}^{\infty}dt\int_{-\infty}^{t}dt'\int_0^{\infty}d\omega\;\;\sin\big[\omega(t-t')\big]\;\mathrm{Im}\big\{G_{zz}(\mathbf{r}-\mathbf{v}t,\mathbf{r}-\mathbf{v}t',\omega)\big\}.$$

Inverting the order of the t and t' integrals, exchanging the t and t' variables, and using reciprocity [Eq. (A3b)], we find

$$\hat{N}_2(\mathbf{r}) = -\,\mathrm{i}\,\frac{8e^2v^2}{\hbar}\int_{-\infty}^{\infty}dt\int_{t}^{\infty}dt'\int_0^{\infty}d\omega\;\;\sin\big[\omega(t-t')\big]\;\mathrm{Im}\big\{G_{zz}(\mathbf{r}-\mathbf{v}t,\mathbf{r}-\mathbf{v}t',\omega)\big\}.$$

The average of these two expressions produces the result

$$\hat{N}_2(\mathbf{r}) = \mathrm{i}\,\frac{4e^2v^2}{\hbar}\int_{-\infty}^{\infty}dt\int_{-\infty}^{\infty}dt'\int_0^{\infty}d\omega\;\;\sin\big(\omega|t-t'|\big)\;\mathrm{Im}\big\{G_{zz}(\mathbf{r}-\mathbf{v}t,\mathbf{r}-\mathbf{v}t',\omega)\big\}, \tag{B1}$$

where the argument of the sine function involves the absolute value of the time difference. Now, we consider the identity [22]

$$\int_0^{\infty}d\omega\;\sin\big(\omega|\tau|\big)\;\mathrm{Im}\big\{\chi(\omega)\big\} = \int_0^{\infty}d\omega\;\cos(\omega\tau)\;\mathrm{Re}\big\{\chi(\omega)\big\}, \tag{B2}$$

which is valid for any response function satisfying $\chi(\omega)=\chi^*(-\omega)$ and having no poles with $\mathrm{Im}\{\omega\}>0$. Applying Eq. (B2) to Eq. (B1) with $\tau=t-t'$ and $\chi(\omega)=G_{zz}(\mathbf{r}-\mathbf{v}t,\mathbf{r}-\mathbf{v}t',\omega)$, and changing the integration variables from t and t' to $z-vt$ and $z-vt'$, we finally obtain Eq. (S14).

REFERENCES

[1] V. Di Giulio, M. Kociak, and F. J. García de Abajo, Optica **6**, 1524 (2019).

[2] This identity is obtained by performing a Taylor expansion of the exponential in $\mathrm{e}^{\mathbf{v}t\cdot\nabla}f(\mathbf{r})=\sum_{n=0}^{\infty}[(\mathbf{v}t\cdot\nabla)^n/n!]f(\mathbf{r})$, such that the right-hand side coincides with the Taylor expansion of $f(\mathbf{r}+\mathbf{v}t)$ around $\mathbf{r}$. From here, we ready verify the equation $f(\mathbf{r})\mathrm{e}^{-\mathbf{v}t\cdot\nabla}g(\mathbf{r})=\mathrm{e}^{-\mathbf{v}t\cdot\nabla}f(\mathbf{r}+\mathbf{v}t)g(\mathbf{r})$ for arbitrary functions $f(\mathbf{r})$ and $g(\mathbf{r})$.

[3] V. Di Giulio and F. J. García de Abajo, New J. Phys. **22**, 103057 (2020).

[4] W. Magnus, Comm. Pure Appl. Math. **VII**, 649 (1954).

[5] Dropping the j subindex for simplicity, we first write $\mathrm{e}^{\beta^*\hat{a}^\dagger-\beta\hat{a}}=\mathrm{e}^{-|\beta|^2/2}\mathrm{e}^{\beta^*\hat{a}^\dagger}\mathrm{e}^{-\beta\hat{a}}$ as a direct consequence of the Baker-Campbell-Hausdorff formula. We now apply the thermal average defined by $\langle\hat{C}\rangle_T=\sum_{n=0}^{\infty}\langle n|\,\hat{C}\,|n\rangle\;p_n$ for any operator $\hat{C}$ in terms of the occupation numbers $p_n=(1-\mathrm{e}^{-\theta})\mathrm{e}^{-n\theta}$ with $\theta=\hbar\omega/k_{\rm B}T$, considering a mode of frequency ω at temperature T. By Taylor-expanding the above exponentials of operators, and noticing that $\langle n|\,(\hat{a}^\dagger)^j\hat{a}^{j'}\,|n\rangle=\delta_{jj'}n!/(n-j)!$ with $j\le n$ (i.e., only $j=j'$ terms survive), we find $\langle\mathrm{e}^{\beta^*\hat{a}^\dagger-\beta\hat{a}}\rangle_T=\mathrm{e}^{-|\beta|^2/2}\sum_{j=0}^{\infty}\big[(-|\beta|^2)^j/j!\big]\,S_j$ with $S_j=\sum_{n=0}^{\infty}p_n\binom{n}{j}$. Then, using the relation[3] $S_j=\bar{n}^j$, where $\bar{n}=1/(\mathrm{e}^{\theta}-1)$ denotes the average population (i.e., the Bose-Einstein distribution), we obtain $\big\langle\mathrm{e}^{\beta^*\hat{a}^\dagger-\beta\hat{a}}\big\rangle_T=\mathrm{e}^{-(\bar{n}+1/2)\,|\beta|^2}$. Finally, this expression becomes $\big\langle\mathrm{e}^{\beta^*\hat{a}^\dagger-\beta\hat{a}}\big\rangle_T=\exp\big\{(1/2)\big\langle(\beta^*\hat{a}^\dagger-\beta\hat{a})^2\big\rangle_T\big\}$ by comparing the exponent to the thermal average of $(\beta^*\hat{a}^\dagger-\beta\hat{a})^2$.

[6] The sum $S_j=\sum_{n=0}^{\infty}p_n\binom{n}{j}=[(1-\mathrm{e}^{-\theta})/j!]\sum_{n=0}^{\infty}n(n-1)\cdots(n-j+1)\,\mathrm{e}^{-n\theta}$ is directly found to satisfy the recursion relation $S_{j+1}=(\bar{n}-j-\partial_\theta)\,S_j/(j+1)$, which trivially admits the solution $S_j=\bar{n}^j$ with $\bar{n}=1/(\mathrm{e}^{\theta}-1)$.

[7] S. Scheel and S. Y. Buhmann, Phys. Rev. A **85**, 030101 (2012).

[8] F. J. García de Abajo and V. Di Giulio, ACS Photonics **8**, 945 (2021).

[9] S. Y. Buhmann, *Dispersion Forces I. Macroscopic Quantum Electrodynamics and Ground-State Casimir, Casimir-Polder and van der Waals Forces* (Springer-Verlag Berlin Heidelberg, Verlag Berlin Heidelberg, 2012).
[10] F. J. García de Abajo, Rev. Mod. Phys. **82**, 209 (2010).
[11] J. D. Jackson, *Classical Electrodynamics* (Wiley, New York, 1999).
[12] M. Born and E. Wolf, *Principles of Optics: Electromagnetic Theory of Propagation, Interference and Diffraction of Light* (Cambridge University Press, Cambridge, 1999).
[13] D. S. Jones, Quart. J. Mech. Appl. Math. **3**, 420 (1950).
[14] In Eq. (S24a), k_z introduces two branch cuts at $k_x = \pm K$ [see Eq. (S25)], but the one at $k_x = -K$ is canceled by the numerator of Eq. (S26).
[15] I. S. Gradshteyn and I. M. Ryzhik, *Table of Integrals, Series, and Products* (Academic Press, London, 2007).
[16] H. C. van de Hulst, *Light Scattering by Small Particles* (Dover, New York, 1981).
[17] F. J. García de Abajo, Phys. Rev. Lett. **102**, 237401 (2009).
[18] H. Nyquist, Phys. Rev. **32**, 110 (1928).
[19] H. B. Callen and T. A. Welton, Phys. Rev. **83**, 34 (1951).
[20] A. A. Abrikosov, L. P. Gorkov, and I. Y. Dzyaloshinskii, *Quantum Field Theoretical Methods in Statistical Physics* (Pergamon Press, New York, 1965).
[21] V. L. Bonch-Bruevich and S. V. Tyablikov, *The Green Function Method in Statistical Mechanics* (North Holland, Amsterdam, 1962).
[22] The functions $\sin(\omega|\tau|)$ and $\mathrm{Im}\{\chi(\omega)\}$ are both odd in ω, so we can write the integral in the left-hand side of Eq. (B2) as $(1/2)\int_{-\infty}^{\infty} d\omega\ \sin(\omega|\tau|)\ \mathrm{Im}\{\chi(\omega)\}$. In addition, by Fourier transforming the sine function, the inverse Fourier transform yields $\sin(\omega|\tau|) = (1/2\pi)\mathrm{P}\int_{-\infty}^{\infty} d\omega' [1/(\omega+\omega') + 1/(\omega-\omega')]\, \mathrm{e}^{-\mathrm{i}\omega'\tau}$, where P stands for the principal value. After making this substitution in Eq. (B2), the ω integral can directly be performed by using the Kramers-Kronig relation $\mathrm{P}\int_{-\infty}^{\infty} d\omega\ \mathrm{Im}\{\chi(\omega)\}/(\omega \pm \omega') = \pi\,\mathrm{Re}\{\chi(\omega')\}$. Finally, noticing the parity of the remaining functions in the integrand and changing ω' to ω, we obtain Eq. (B2).
[23] C. W. Johnson, A. E. Turner, and B. J. McMorran, Phys. Rev. Research **3**, 043009 (2021).
[24] C. W. Johnson, A. E. Turner, F. J. García de Abajo, and B. J. McMorran, Phys. Rev. Lett. **128**, 147401 (2022).

SUPPLEMENTARY FIGURES

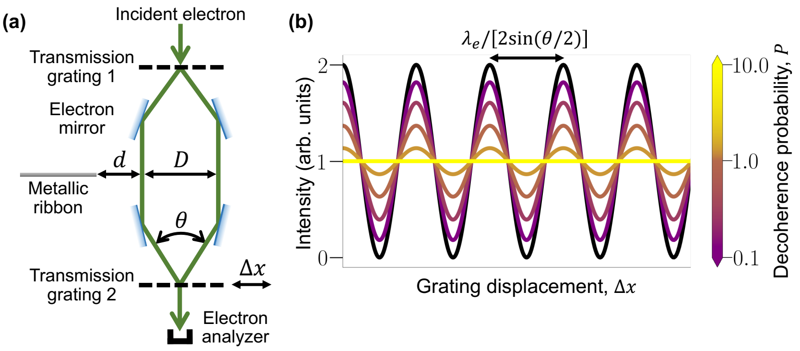

FIG. S1: **A possible configuration for measuring electron decoherence with large inter-path separations.** (a) Electron optics scheme incorporating a transmission grating that splits the incident beam into two paths; two upper electrostatic mirrors that redirect the paths such they run parallel to each other with a separation D at the position of the specimen (e.g., a wide metallic ribbon); two subsequent mirrors that make the paths converge; a second movable transmission grating that mixes the two paths into a single Bragg transmission direction; and an electron detector. The second grating must have a period $\lambda_e/\sin(\theta/2)$ determined by the angle θ between the two convergent paths and the electron wavelength λ_e, such that Bragg scattering combines both of them along a direction normal to the grating. The electron signal oscillates with the lateral displacement of the grating Δx, provided the two paths maintain some degree of coherence. (b) Interference pattern as a function of grating 2 displacement Δx for different values of the decoherence probability produced by radiative coupling mediated by a metallic half-plane under the configuration of (a) (see specific examples in Figs. 1 and S2 below). The interference patterns have a period $\lambda_e/[2\sin(\theta/2)]$ in Δx. Gratings similar to those used in previous studies [23, 24] would fit in the current scheme.

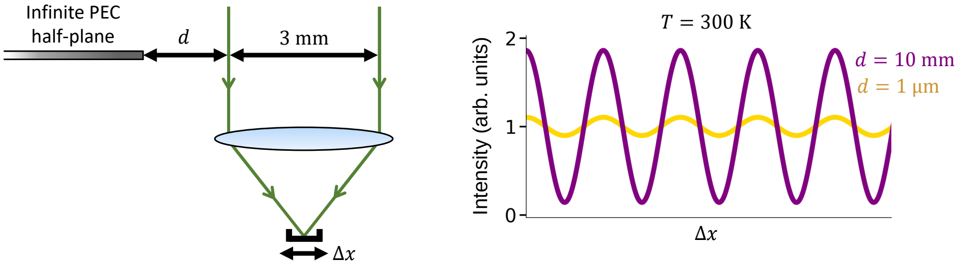

FIG. S2: **Depletion of fringe visibility in a feasible experimental scenario.** We consider a configuration similar to Fig. 1c in the main text, where a two-path electron interacts with a perfect-electric-conductor (PEC) half-plane at a temperature of 300 K and undergoes a degree of decoherence that is observed through the visibility of the interference fringes obtained as a function of transverse position Δx (or deflection angle) when mixing both paths at a detector (see also Fig. S1). Here, we fix the inter-path separation to 3 mm and consider two different electron-edge distances: $d = 1\ \mu$m and 10 μm. A substantial reduction in fringe visibility is predicted for the shortest d.

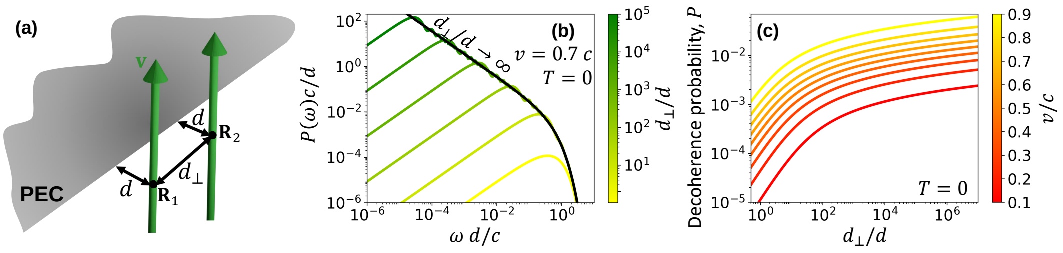

FIG. S3: **Two-path electron decoherence by a half-plane at zero temperature in a laterally displaced arrangement.** (a) System under consideration, consisting of a single electron split into a two-path spatial superposition and passing close and perpendicularly to a perfectly conducting half-plane at a distance d from the edge with a lateral separation $d_\perp$ [beam path positions $\mathbf{R}_1 = (d, 0)$ and $\mathbf{R}_2 = (d, d_\perp)$]. (b) Universal plot of the spectrally resolved decoherence probability for various $d_\perp/d$ ratios (color curves), approaching a divergent profile in the $d_\perp \gg d$ limit (black curve). We consider an electron velocity $v = 0.7\,c$ and normalize the frequency and the probability using the distance d. (c) Decoherence probability as a function of $d_\perp/d$ for different electron velocities (see color scale).

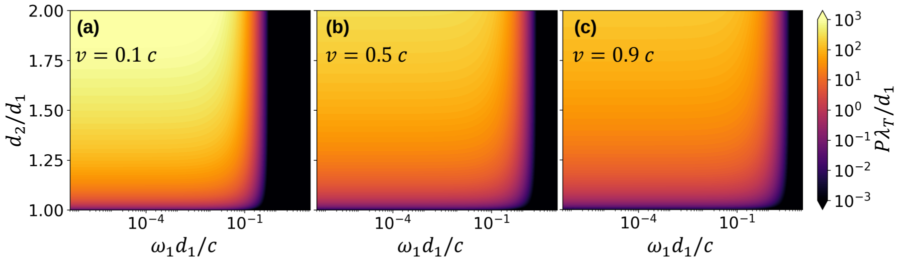

FIG. S4: **Spectral decomposition of the decoherence probability by a half-plane at finite temperature.** We consider the configuration of Fig. 2a in the main text with $d_\perp = 0$ and present results for three different electron velocities in panels (a)-(c) (see labels).

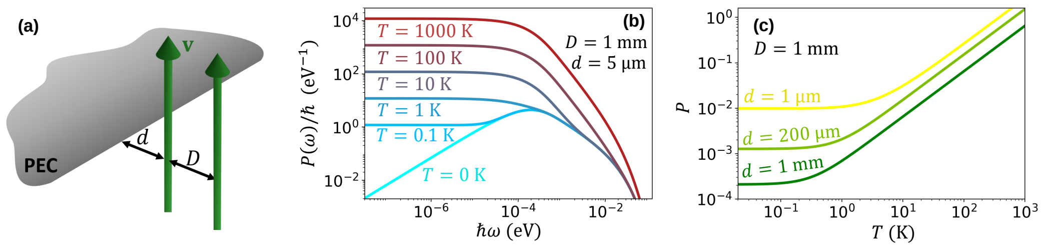

FIG. S5: **Onset of temperature effects in electron decoherence.** (a) We consider a two-path electron beam passing near a metallic half-plane with electron-edge and inter-path separations d and D, respectively. (b) Spectral decomposition of the decoherence probability for $d = 5\ \mu$m, $D = 500\ \mu$m, and different temperatures in the $T = 0 - 1000$ K range. (c) Temperature dependence of the decoherence probability for $D = 500\ \mu$m and various values of d.

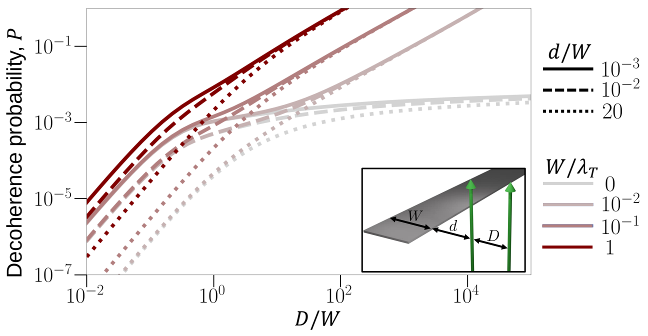

FIG. S6: **Finite size effects on the decoherence probability.** Similar to Fig. 4 in the main text, but with the decoherence probability P plotted as a function of the inter-path separation D normalized to the ribbon width W. The electron beam is prepared in a two-path superposition state (inter-path distance D) and interacts with a ribbon of width W. The path–ribbon distances are d and $d + D$. We present results for different values of the d/W and W/λ_T ratios (see the legend on the right).
